# Supplementary material for: Patient journey following lumbar spinal fusion surgery (FuJourn): A multicentre exploration of the immediate post-operative period using qualitative patient diaries
Source: PLoS One. 2020 Dec 1;15(12):e0241931. doi: 10.1371/journal.pone.0241931 (PMC7707593; doi:10.1371/journal.pone.0241931)
Supplement: S1 Appendix — (PDF) [file pone.0241931.s001.pdf]

UNIVERSITY OF BIRMINGHAM

S1 Appendix: Sample diary

Patient Diary for week ending: Sunday _ _ / _ _ / _ _

Participant number:

The diary aims to record your journey following your spinal surgery by tracking your experiences on a weekly basis. The diary is broken down into a number of sections. These sections are intended to act as a prompt in helping you to record how you have felt and what you have experienced during the past week. Please respond freely and honestly as there are no right or wrong answers. If a section is not relevant to you this week, please move onto the next prompt.

Prompt	***Diary entry***
Feelings about your progress this week	
Feelings about your recovery so far	
Level of motivation	
Symptoms you have experienced over the past week	
Medications you have taken this week	
Healthcare appointments this week	

Rehabilitation progress this week<br>- Your ability to exercise and do activity this week<br>- Return to work / activities / hobbies as relevant to you	
Positive thought(s) this week	
Negative thought(s) this week	
Most significant/memorable moment this week<br>(may be positive or negative)	
Please describe how this week has been compared to last week	

Please record any other comments you would like to make regarding how you have felt or your experiences over the past week?

Thank you for your completing the diary this week – your help is very much appreciated.
