# Supplementary material for: Patient journey following lumbar spinal fusion surgery (FuJourn): A multicentre exploration of the immediate post-operative period using qualitative patient diaries
Source: PLoS One. 2020 Dec 1;15(12):e0241931. doi: 10.1371/journal.pone.0241931 (PMC7707593; doi:10.1371/journal.pone.0241931)
Supplement: S2 Appendix — (PDF) [file pone.0241931.s002.pdf]

S2 Appendix: Description of themes

Diary entry: P1

**Participant: P1**	**Entry**				**Categories/Themes**			
**Prompt**	**Week 1**	**Week 2**	**Week 3**	**Summary**	**Week 1**	**Week 2**	**Week 3**	**Combined themes**
Feelings about your progress this week	Good progress until Friday evening then bit of a rough weekend	Really mixed week in terms of progress. It changes from hour to hour but is moving in the right direction	I feel mainly positive about this week's progress	Good progress, mixed week in terms of progress but remaining positive	Fatigue<br>Energy (less)	Positive<br>Movement (+)	Uplifted<br>Positive<br>Motivation	Positive<br>Motivation<br>Energised (+)
Feelings about your recovery so far	Probably better recovery so far in general than I had expected	I am now walking around without crutches with the exception of stairs	I think I'm far better than I expected to be at this stage	Better recovery than expected, walking unaided	Empowerment<br>Control<br>Meaningful	Control (+)<br>Satisfying (+)<br>Regaining	Belief<br>Control (+)<br>Hope (+)	Control (+)<br>Empowerment<br>Belief<br>Hope (+)
Level of motivation	I was really motivated to leave hospital asap which in hindsight was a little bit to early	I am now walking around without crutches with the exception of stairs	Mainly good... I have started looking forward to getting back into some sort of exercise in the future	Walking unaided with the exception of stairs, contemplate taking up some exercise	Drive<br>Incentive (+)<br>Hasty	Control (+)<br>Self-belief<br>Limitation	Self-efficacy<br>Positive	Self-efficacy<br>Positive<br>Control<br>Drive
Symptoms you have experienced over the past week	Nothing unexpected really....some pain, a pressure sore on my lower back/bottom, tiredness.	I have had chills and sweats, severe pain and tiredness at times through the week but also good moments. I have also had sciatic pain in my leg	I have had quite a bit of leg pain (sciatic) this week, and also pain in the left side of my groin. The groin pain could be because of the bone graft I suppose.	Experienced some leg pain sometimes severe associated with bone graft and potentially sciatica	Physical<br>Mood	Pain<br>Physical<br>Sustained	Pain<br>Symptoms<br>Fatigue	Pain<br>Physical<br>Fatigue<br>Symptoms
Medications you have taken this week	200mg Sertraline.	200mg Sertraline.	200mg Sertraline.	Adherence to prescriptions	Adherence<br>Awareness	Adherence<br>Awareness	Adherence<br>Awareness	Adherence<br>Awareness

Participant: P1	Entry				Categories/Themes			
**Prompt**	**Week 1**	**Week 2**	**Week 3**	**Summary**	**Week 1**	**Week 2**	**Week 3**	**Combined themes**
	2,700mg Gabapentin. 240mg codeine. Paracetamol x ? Morphine x ? and 25mg amitriptyline PER DAY	2,700mg Gabapentin. 240mg codeine. Paracetamol x ? and 25mg amitriptyline PER DAY	2,700mg Gabapentin. 240mg codeine. Paracetamol x ? and 25mg amitriptyline PER DAY					
Healthcare appointments this week	In hospital until Friday evening	District nurse came out to dress a pressure sore on my lower back/bottom	none	Adequate access to health care professionals	Awareness<br>Adherence	Access<br>Symptoms	None	Awareness<br>Access<br>Adherence
Rehabilitation progress this week<br>- *Your ability to exercise and do activity this week*<br>*Return to work / activities / hobbies as relevant to you*	I managed to use a walking frame to walk a few yards on 11th Oct. and walked down the corridor and a few steps using crutches on 12th. On 13th & 14th I was at home were although I felt I had left hospital a bit early I did manage to get around the house and use the stairs using my crutches	I am able to get around easier as the week has progressed and I have been for a couple of short walks approximately 500metres long	I have hardly used my crutches this week	Able to get around easier with minimal reliance on crutches and walking frame	Positive (+)<br>Self-motivation	Belief<br>Self-motivation<br>Self-efficacy	Belief<br>Self-efficacy<br>Uplifted<br>Control	Self-efficacy<br>Control<br>Self-motivation<br>Uplifted
Positive thought(s) this week	After speaking to my surgeon, I believe my back will be better	I am looking forward to being able to play with my children again	I sometimes think I'm going to make a full recovery with no back pain	Belief that back pain will improve and potentially	Belief<br>Positive (+)	Motivation<br>Goal<br>Ability	Belief (+)<br>Goal<br>Motivation	Belief<br>Positive<br>Goal<br>Motivation

Participant: P1	Entry				Categories/Themes			
**Prompt**	**Week 1**	**Week 2**	**Week 3**	**Summary**	**Week 1**	**Week 2**	**Week 3**	**Combined themes**
	than it was before.			making a full recovery				
Negative thought(s) this week	I can't think of a negative thought I had this week to be honest.	When the pain gets bad I do sometimes wonder if it's going to get better but in general more positive than negative thoughts	sometimes think I'm not going to make a full recovery and I'm still going to have a back problem	Belief that recovery may not be attainable but staying positive nevertheless	Mood<br>Self-control	Mood<br>Self-belief	Belief (-)<br>Anxiety	Belief<br>Anxiety
Most significant/memorable moment this week (may be positive or negative)	On 12th walking with crutches down the corridor and up/down a few stairs felt really good.	Walking around the ground floor of the house without crutches	Had some really strong pain in legs this week	Positive attitude and progress with movement starting with crutches down corridor followed by walking without crutches	Self-control<br>Belief	Belief<br>Control<br>Speed	Anxiety	Control<br>Belief<br>Emotion
Please describe how this week has been compared to last week	On 12th walking with crutches down the corridor and up/down a few stairs felt really good.	Much better than last week in all areas	Definitely getting easier to get around the house	Generally good progress with movement	Self-control<br>Belief	Control<br>Satisfaction<br>Self-efficacy	Control<br>Belief<br>Movement	Self-efficacy<br>Belief<br>Satisfaction
Please record any other comments you may like to make regarding how you have felt or your experiences over the past week?	I think I made good progress but maybe should have stayed in hospital for a day or 2 more.	No data recorded	No data recorded	Good progress but recognise the need to allow time to recover adequately				Positive

Diary entry: P2

Participant: P2	Entry					Categories/Themes				
**Prompt**	**Week 1**	**Week 2**	**Week 3**	**Week 4**	**Summary**	**Week 1**	**Week 2**	**Week 3**	**Week 4**	**Combined themes**
Feelings about your progress this week	Came home Friday night slept very tired. Sat I was ok, improved Sunday	I feel I have progressed slightly better. I can move around the house well with crutches sometime without crutches, but muscles hurt when I do too much	I am really chuffed this week. I feel I have improved 80%	Very happy with progress	Felt significant improvement in movement but painful when overdoing	Energy (-) Fatigue	Energy (+) Movement (-)	Speed (+)	Speed Energy	Energised Speed Fatigue
Feelings about your recovery so far	I feel ok. I Know I have to take it slow	It's slow recovery, I miss walking with my dogs, but they are staying close by. Trying not to get frustrated	So far so good, I still have good days and bad days more goal/excellent today. But had one awful day.	Still having good and bad times. I'm getting frustrated that I can't do more	Slow recovering, aware that it is a lengthy process. Frustrated about reduce physical activity	Awareness	Frustration	Frustration Anxiety	Frustration Anxiety	Frustration Anxiety Awareness
Level of motivation	Good level	I have ups and downs moments as I know not to get down too much	Very motivated this week started week with one crutch now no crutches. 10 for motivation 1 big bid 10 goals	Good level I'm enjoying walking even in the rain	Reasonable level of motivation. Setting out goals	Positive (+)	Speed (-)	Energy (+) Motivation (+)	Energy Movement	Motivation Speed Energy
Symptoms you have experienced over the past week	Graft site pain numbness of left thigh.	Stinging each down left leg. Bladder hurting	Ache in back but for muscles still left leg ache	Ache in my back this week and tightness	Mostly back and left leg aches. Tightness	Pain Anxiety	Pain	Pain	Pain	Pain Anxiety

Participant: P2	Entry					Categories/Themes				
**Prompt**	**Week 1**	**Week 2**	**Week 3**	**Week 4**	**Summary**	**Week 1**	**Week 2**	**Week 3**	**Week 4**	**Combined themes**
	One shooting pain down left foot on Saturday night. Not doing very well.	alternating (muscles nerve repair)	and more pain numb over left quad	around stomach incision side leg pain	around incision site					
Medications you have taken this week	Oral morphine, paracetamol, ibuprofen, lacxillo, cycline	Oral morphine, pregan, 75 mg paracetamol, ibuprofen, diazepam	Pregance 150mg for leg symptoms, paracetamol;l 4x a day ibuprofen x3 oral morphine twice before bed this week	Paracetamol, ibuprofen, pregnv twice or more at night diazepam to help sleep and leg pain	Appears to be taking medication dependent on the level of pain experienced	Threshold	Pain	Pain<br>Sleep	Pain<br>Sleep	Pain<br>Sleep<br><br>**Dependent on pain and sleep pattern**
Healthcare appointments this week	None	None	Monday 26th sutures out nice to see them gone	None	Limited access to healthcare	None	None	Positive	None	Positive<br>Minimal
Rehabilitation progress this week<br>- *Your ability to exercise and do activity this week*<br>*Return to work / activities / hobbies as relevant to you*	Stairs and walk around house very slowly, not a lot at once	Crutches around house	Walked to the supermarket on Thursday with parents. 2xwalk 10 mins, Friday, Sat, Sun stairs on won as walking normally. Bend down using legs not back to pick up crutch I dropped body upright	Walk twice a day need to stand up a lot this week. Did some drawing	Improved movement. More stamina and well enough to take up a hobby.	Energy (-)<br>Motivation	Energy<br>Movement	Speed<br>Movement<br>Energy (+)	Energy<br>Motivation<br>Stamina	Energy<br>Motivation<br>Stamina
Positive thought(s) this week	Mostly all positive	Bowels moving again. Losing	Good to see sutures gone nice visually. Good to go	Improving walking well	Weight loss, skin visually good/. Improved	Positive	Anxious	Positive	Energy (+)	Positive<br>Anxiety<br>Energy

Participant: P2	Entry					Categories/Themes				
**Prompt**	**Week 1**	**Week 2**	**Week 3**	**Week 4**	**Summary**	**Week 1**	**Week 2**	**Week 3**	**Week 4**	**Combined themes**
		a lot of weight	for a walk good mentally		mental health wellbeing.					
Negative thought(s) this week	Need to poo	Painful at night, finding it hard to get to sleep gone midnight. Hard in shower need help	Want to do more hard when use to walk mountains every weekend. Friday hard feet poorly	Painful to sit down hard to get to sleep	Painful at night, disturbed sleep. ADL affected. Frustrated cannot resume normal activity	Anxious	Anxious Energy	Pain Depression	Pain Sleep	Pain Energy Depression Sleep
Most significant/memorable moment this week (may be positive or negative)	Having the surgery positive	I can feel my feet properly again. Even itchy slow bits	Sutures gone, yeppi make you feel amazing even though it's just the sutures	Being able to move up and down stairs very well	Improved movement. Able to walk up/down stairs. Skin visually better	Positive	Movement Feeling	Happy	Energy	Energy Positive
Please describe how this week has been compared to last week	1st walk amazing	I'm out of hospital so at home when I do too much I can tell the next day. So I have to pace myself	80% better week than last week I can walk around unaided at home+10 mins walk	Improvement again, good in a positive way	Learning to pace not to overdo any activity. Almost fully able to move normally	Movement (+)	Anxiety Awareness Energy	Self-efficacy Movement	Energy Optimistic	Anxiety Energy Optimistic Self-efficacy
Please record any other comments you may like to make regarding how you have felt or your experiences over the past week?	Unconfy to sleep when I am on my side my stomach pulls. But I think this is because my stomach is very bloated	At the beginning of the week I was doing too much and found it hard and painful for a few days later. I must slow down and not rush around be patient, I	Monday was amazing having sutures out. Friday was an awful day I felt poorly I hurt I felt like I been hit by a bus maybe walk around supermarket to much than I	I'm struggling with socks and shoes as I can't bend have to sit up straight or feel pain in back. Shooting and sweats I've had one moment of	Disturbed sleep pattern. Sometime emotional. Better days. Need to learn to pace movement	Sleep Energy	Pain Fatigue	Pain	Sleep Emotion	Pain Fatigue Energy

Participant: P2	Entry					Categories/Themes				
**Prompt**	**Week 1**	**Week 2**	**Week 3**	**Week 4**	**Summary**	**Week 1**	**Week 2**	**Week 3**	**Week 4**	**Combined themes**
		miss stretching, Pilates and walking. I keep popping outside for fresh air to stop my cabin fever	did a lot of walking at home. But better a Saturday it is hard to not help and feel harder mentally. But back feels great! Legs have improved still strange numb, stinging sensation on left thigh wound on abdo has gone.	crying not sure why but felt much better after						

Diary entry: P3

Participant: P3	Entry					Categories/Themes				
**Prompt**	**Week 1**	**Week 2**	**Week 3**	**Week 4**	**Summary**	**Week 1**	**Week 2**	**Week 3**	**Week 4**	**Combined themes**
Feelings about your progress this week	I can walk well with the aid of a walking stick. Extremely tired	Still walking well but I find if I go out for a walk I am lifeless with extreme tiredness the next day	Doing well. I can now walk to the supermarket and back without a break still with walking stick	Feeling proud, managed to walk into town and back with many stops took about 4 hours but I did it	Periodic feeling of extreme tiredness. Motivated to walk	Energy (-) Motivation	Motivation Fatigue (+)	Movement Energy (+)	Positive Energy	Positive Energy Motivation
Feelings about your recovery so far	I am sore and uncomfortable but not	The medication keeps the pain	Feeling positive but still so tired I	Still positive	Realistic but positive. Still feeling	Pain (+) Anxiety	Fatigue (+) Pain	Positive (+) Energy	Positive	Positive Fatigue Anxiety

Participant: P3	Entry					Categories/Themes				
**Prompt**	**Week 1**	**Week 2**	**Week 3**	**Week 4**	**Summary**	**Week 1**	**Week 2**	**Week 3**	**Week 4**	**Combined themes**
	expecting too much too soon	at bay which is helping me to go out for a walk. Still extreme tiredness	can only go out every other day		extreme tiredness					
Level of motivation	Motivated to walk about because I know it helps recovery	I managed to go to my friend's 50th on Saturday. Didn't stay long but it felt good to be able to do that	Motivation is good on the days I go out. On the days I'm at home I'm so tired that I don't get distressed	Motivation is still good but extreme tiredness is still there	Motivated but extreme tiredness at times	Motivation	Positive Energy	Motivation	Motivation Fatigue	Motivated Fatigue Energy
Symptoms you have experienced over the past week	Pain in my back. Got an infection in my wound and went to the walk in centre, they cleaned it and gave antibiotics. Left hospital numb and tingly right top leg, it's still there.	Infection still there so a second course of antibiotics pain there if I don't take medication on time. Right top leg tingly and numb.	Numb lower back to my bum cheek. Infection cleared up but wound on my back not healed. Tummy wound doing well. Right leg still cold and tingly	Still numb feeling in left lower back and bum cheek. Right top leg cold numb and tingly	Wound infection, feeling numb and tingly.	Pain Symptoms	Symptoms Pain	Pain Anxiety	Pain Anxiety	Pain Anxiety
Medications you have taken this week	Oxycodin, senna, naproxen, antibiotics, amytripline, mirtapazapine, pregabalin	Tramadol, antihistamine, naproxen, amytripline, pregbalun, paracetamol, senna, antibiotics	Tramadol, antihistamine, paracetamol, senna, naproxen, pregbalum, antibiotics, amytryptiline, mirtazapen	Tramadol, paracetamol, antihistamine, naproxen, amitripline, pregabalum, senna mirtazapen	Appears to be dependent of level of pain exeperienced	Symptoms	Pain	Pain Bowel	Pain	Pain

**Participant: P3**	**Entry**					**Categories/Themes**				
**Prompt**	**Week 1**	**Week 2**	**Week 3**	**Week 4**	**Summary**	**Week 1**	**Week 2**	**Week 3**	**Week 4**	**Combined themes**
Healthcare appointments this week	Walking centre on Thursday, Friday and Sunday	Walk in centre on Tuesday and Friday. All but 2 stitches removed	Walking centre on Tuesday removed last 2 stitches	None	Access due to infection primarily	Access	Access	Access	None	Access
Rehabilitation progress this week<br>- *Your ability to exercise and do activity this week*<br>*Return to work / activities / hobbies as relevant to you*	On Friday I took my first trip out to the supermarket with my son and I used a walking stick. Walked there stopped for hot chocolate and then walked home. Wasn't too slow I felt good	Walked very other day now get in the shower without help	Still walking every other day	Walking to town and back everyday	Motivated to walk, able to set target	Motivated Support	Motivated Positive	Positive (+)	Energy	Positive Motivated Energy Support
Positive thought(s) this week	I'm going home	I'm really lucky to have a good support network of family and friends around me	All stitches now removed	I'm continuing to walk further and rest when needed	Thought of going home, family support and rate of healing	Positive	Support	Positive	Energy Positive	Energy Positive Support
Negative thought(s) this week	Scared to go home	Infection in back more medication stitches to stay in	No data	No data	Lack of support and fear wound won't heal and recovery may take longer	Anxiety	Positive (-)	No data	No data	Anxiety Symptoms
Most significant/memorable moment this week(may	Walked to Sainsbury with my son	I got to my friend's 50th party for a	No data	Seemed to have gone very quick	Good support mechanism	Energy Support	Energy Movement (+)	No data	Support Positive	Energy Support Movement

Participant: P3	Entry					Categories/Themes				
**Prompt**	**Week 1**	**Week 2**	**Week 3**	**Week 4**	**Summary**	**Week 1**	**Week 2**	**Week 3**	**Week 4**	**Combined themes**
be positive or negative)		couple of hours			(family & friend)					
Please describe how this week has been compared to last week	A bit scary because I am at home but I've got plenty of family and friends around me	Feels a bit easier to move about	Not too dissimilar to others walking and resting when needed	I still being spoiled with care from family and friends	Reassurance of family support	Anxiety<br>Support	Movement (+)<br>Positive	Movement<br>Energy	Support	Anxiety<br>Support<br>Positive
Please record any other comments you may like to make regarding how you have felt or your experiences over the past week?	My children bother and my friends have made my return home very easy. I'm very lucky. I live alone	My back feels sore, tight and itchy my tummy feels ok	No data	No data	Family support. Back and stomach feels sore and itchy	Support	Pain			Support<br>Pain

Diary entry: P4

Participant: P4	Entry					Categories/Themes				
**Prompt**	**Week 1**	**Week 2**	**Week 3**	**Week 4**	**Summary**	**Week 1**	**Week 2**	**Week 3**	**Week 4**	**Combined themes**
Feelings about your progress this week	Relatively slow due to the fact that I was released from hospital in January 2018. Returned home taking the medication that was prescribed by the hospital. At the moment I am walking	The progress this week has been very good. At the end of this week I hope to start driving obviously with somebody else in the car with me.	Progress this week has been good. Still got the slight numbness to the left foot but seems to be getting better. So we'll see how it goes	Progress this week has been hindered by the weather. Haven't been able to get out as much as I would have liked to due to the uneven ground and I don't want to	Satisfactory progress, feeling positive	Positive (+)<br>Energy	Positive (+)<br>Speed	Positive (+)<br>Anxiety<br>Pain	Movement<br>Anxiety	Positive<br>Energy<br>Pain<br>Movement<br>Anxiety

Participant: P4	Entry					Categories/Themes				
**Prompt**	**Week 1**	**Week 2**	**Week 3**	**Week 4**	**Summary**	**Week 1**	**Week 2**	**Week 3**	**Week 4**	**Combined themes**
	around and have managed to negotiate 2 flights of stairs.	I feel positive that I can do it but that will be updated in next weeks diary.	during the week.	fall and put myself back.						
Feelings about your recovery so far	Recovery so far has been as expected or maybe I wasn't fully aware of the amount of surgery I undertook.	This week has been extremely good, walking further and climbing stairs as normal. Been shopping and out for meals so onwards and upwards.	Recovery on week 4 seems absolutely fine. Stiches healed. Slight discomfort on the actual wound area but I think that is bruising that im feeling.	Recovery is going in the right direction. Left foot is slowly getting movement back in the big toe due to a different exercise im undertaking.	Awareness of recovery, positive attitude	Expected Awareness	Positive (+) Energy	Positive (+) Energy	Movement Positive (+)	Positive Energy Expected
Level of motivation	To get back to a better standard of living as in manoeuvrability.		Motivation is still high. Pushing myself past what I was doing last week but obviously still taking it slowly. But progress is going in the right direction.	Motivation is still high.	Driven	Drive	No data	Energy Positive (+)	Positive (+)	Positive Drive Energy
Symptoms you have experienced over the past week	Slight less to left foot mainly the toe	This week the same as the previous	No new symptoms. Just	No new symptoms this week apart from	Restricted movement,	Movement	Anxiety Hope Pain	Pain	Speed	Pain Speed Anxiety

Participant: P4	Entry					Categories/Themes				
**Prompt**	**Week 1**	**Week 2**	**Week 3**	**Week 4**	**Summary**	**Week 1**	**Week 2**	**Week 3**	**Week 4**	**Combined themes**
	area. I think my body is still quite swollen and some of the nerves that lead down to the foot are still being compressed – in my opinion!	2 weeks, I still have concerns with the my left foot with the numbness in the area of the big toe. This is making me slightly concerned again however I have been told my several members of the medical profession that I am only at the end of the third week it is still extremely early for things to be fully operational.	discomfort in the left foot – slight numbness but it's not getting worse.	progress with the big toe in the left foot which appears to be getting better.	pain discomfort					Hope
Medications you have taken this week	The medications that I have been prescribed after day 4 of being at home I stopped taking the stronger painkiller and discontinued	No medications all this week.	Too one days worth of paracetamol morning and evening dose one day mid week because I	No medication taken this week.	Adherence to medication in view of reducing intake	Adherence	Management	Management	Management	Management Adherence

Participant: P4	Entry					Categories/Themes				
**Prompt**	**Week 1**	**Week 2**	**Week 3**	**Week 4**	**Summary**	**Week 1**	**Week 2**	**Week 3**	**Week 4**	**Combined themes**
	taking the paracetamol and the senacot to obviously keep my bowels open.		getting some internal pains but that's all stopped so no medication apart from that one day.							
Healthcare appointments this week	I have no healthcare appointments this week.	Returned to the GP practice nurse this week to have the dressing removed and she is of the opinion that the wound is 95% healed, closed up and can leave the dressing off and im able to bathe.	Returned to practice nurse and all looking good with the wound so looking forward to a longish soak in the bath.	No appointments this week.	Managemen t of wound effectively	Awareness	Hope	Positive (+)	Awareness	Awareness<br>Management<br>Hope<br>Positive
Rehabilitation progress this week<br>- *Your ability to exercise and do activity this week*<br>*Return to work / activities / hobbies as relevant to you*	I have been out onto the street and walked to the local shop but at a greatly reduced speed. The exercise I have been doing within the house is just walking about, walking up and down the stairs and generally just	Same as last week. Just walking further and climbing stairs in house more often.<br>I'm retired so no work to return to. I'm still not able to do any activities as	Still exercising as much as I can. Stated to use a leg exercise machine to strengthen the muscles in the legs and thighs. But other than that still walking as	Exercising indoors this week as snow has put a dampener on exercising outdoors and safely.<br>No work as I'm retired.	Pacing activities, awareness of timescale	Hinder	Speed<br>Movement	Awareness<br>Positive (+)	Positive (+)	Positive<br>Hinder<br>Awareness<br>Movement

Participant: P4	Entry					Categories/Themes				
**Prompt**	**Week 1**	**Week 2**	**Week 3**	**Week 4**	**Summary**	**Week 1**	**Week 2**	**Week 3**	**Week 4**	**Combined themes**
	keeping as mobile as I can.	they probably would be too strenuous and they involve lifting things.	much as I can.							
Positive thought(s) this week	Motivation to get myself fully or as fully fit as I can for a family holiday at the end of April.	Looking forward to being able to drive my car at the end of this week or the beginning of next week.	Having a bath – what a luxury.	Being able to move the left foot and toe slightly better than previously. Some improvement is better than no improvement.	Motivated to get fully fit	Motivation	Positive (+)	Positive (+)	Movement	Positive Movement Motivation
Negative thought(s) this week	Not many. My only concern is the slight differential pain between the left and the right foot. I have spoke to my consultant's secretary who has spoken to to my consultant who has reassured me at this stage as it's the very early recovery period, that this numbness that I have got is possibly down to parts of nerves still being trapped by	No negative thoughts.	No negative thoughts.	Nothing really apart from the snow putting a dampner on things.	Maintaining positive thoughts	Positive (+)	Positive (+)	Positive (+)	Positive (+)	Positive

Participant: P4	Entry					Categories/Themes				
**Prompt**	**Week 1**	**Week 2**	**Week 3**	**Week 4**	**Summary**	**Week 1**	**Week 2**	**Week 3**	**Week 4**	**Combined themes**
	the swollen area in the main back. Can't really say about any negative thoughts this week as its pointless being negative when you've got to look forward to the positive things									
Most significant/memorable moment this week (may be positive or negative)	Was going out for a meal to a restaurant and feeling like I was being accepted back into the community. Silly thought but it was nice to get out.		Having a bath. Driving a short distance in the car. Obviously having my wife next to me in the passenger seat just in case.	Nothing really apart from the wife returning from holiday in 2 days' time.	Some normality	Speed Support	No data	Reassurance	Positive	Positive Speed Reassurance
Please describe how this week has been compared to last week	This week has been totally different to last week as I hadn't had the operation. I'm in pain, well was in pain so its hard to give feedback on this but hopefully next week once this first week has	This week has been an improvement on last week so again, onwards and upwards.	Good. Things seem to be going in the right direction which is good so onwards and upwards.	Has been slightly better in some ways than last week as that has been to do with the progress made with regards to the toe/foot.	Feeling pain periodically. Acknowled ged reasonable progress	Positive (+) Pain	Positive (+)	Positive (+)	Progress Energy	Energy Positive (+) Pain

Participant: P4	Entry					Categories/Themes				
**Prompt**	**Week 1**	**Week 2**	**Week 3**	**Week 4**	**Summary**	**Week 1**	**Week 2**	**Week 3**	**Week 4**	**Combined themes**
	settled down I'll be able to be positive on how the next week compared to this week.									
Please record any other comments you may like to make regarding how you have felt or your experiences over the past week?	The main thing at the moment is that its still hard to report but on what I can feel the pain I had before the operation is no longer present in the lower back and the legs. I still have pain obviously which is no the same sort of pain so I'm hoping that the problems I did have, have been rectified by the surgery.									Positive Pain

Diary entry: P5

Participant: P5	Entry					Categories/Themes				
**Prompt**	**Week 1**	**Week 2**	**Week 3**	**Week 4**	**Summary**	**Week 1**	**Week 2**	**Week 3**	**Week 4**	**Combined themes**
Feelings about your progress this week	Mixture of emotions. Ranging from euphoria to anxiety. Lots of feelings about impact and outcomes of surgery on activities of daily living and work commitments	Up and down. There have been some highs and lows but overall I am feeling positive with regard to the outcome of the surgery	There is definite progress. I am still having good days and less good days in terms of how I feel physically and emotionally, but overall definite improvement in confidence with mobility and other daily activities.	Overall Good. Lots of milestones achieved,	Mixed feelings	Happy Anxiety	Positive (+)	Confident Movement	Energy	Positive Energy Anxiety
Feelings about your recovery so far	Mainly positive with some caution due to changes in geography of pain and discomfort in the post operative period. Cautiously optimistic. I appreciate it is going to be a long rehabilitation process and I am slowly accepting that fact.	Recovery is slow and steady and I maybe hadn't quite prepared myself for that. However this week has resulted in me becoming much more independent with regard to the daily activities of living, with some obvious restrictions with regard to food preparation. I am assisting with daily household	Pleased and grateful and ready to keep working hard with the recovery phase.	That hard work seems to be paying off. Gently increasing mobilisation and able to perform more household tasks but still some way to go yet. Still some residual issues	Steady recovery	Frustration	Energy	Energy	Movement Energy	Energy Cautious Positive

Participant: P5	Entry					Categories/Themes				
**Prompt**	**Week 1**	**Week 2**	**Week 3**	**Week 4**	**Summary**	**Week 1**	**Week 2**	**Week 3**	**Week 4**	**Combined themes**
		duties as far as my surgery currently allows, which in turn helps to foster feelings of positivity. Mild degree of frustration with regard to not feeling able to undertake household chores. I feel grateful and blessed that I have a fantastic family and friends who are on hand to assist me as necessary..								
Level of motivation	I am a very motivated person ordinarily but symptoms such as post operative surgical pain, exhaustion and nausea can impact on that motivation. Trying to keep a bit of a routine with regard to ensuring adequate mobility is	My level of motivation is quite high. I am trying to establish some sort of routine to the day and ensure that the balance between mobilisation and rest periods is sensible to assist in the post operative recovery. I need to remind myself that it is still	Excellent this week. I had a goal to attend a work presentation for an award my team had achieved. It was the culmination of two years of my work life so I was determined to be there with my team. This involved me getting fully	Overall positive. Still experiencing marked fatigue one or two days in the week. As I've always been borderline anaemic with a lower blood pressure, it could be linked, but	Balancing mood and positivity	Mood Motivation	Positive (+)	Goal Positive(+)	Fatigue	Goal Fatigue Positive Motivation

Participant: P5	Entry					Categories/Themes				
**Prompt**	**Week 1**	**Week 2**	**Week 3**	**Week 4**	**Summary**	**Week 1**	**Week 2**	**Week 3**	**Week 4**	**Combined themes**
	achieved whilst balancing against the fear of 'overdoing it' which could impact on healing process.	'early days'. I am using this time positively to undertake tasks that I have been putting off but am able to complete at this point in my recovery.	dressed, travelling in a car and waiting for the ceremony to start in a public place. (Slight feeling of vulnerability but glad to be outside and back in the real world).	not bad enough for me to see GP. I still feel very much in the ongoing recovery phase.						
Symptoms you have experienced over the past week	Unusual geographical changes of areas of pain/ discomfort in the right limb, groin and surgical site. There is obvious improvement, but localised tenderness makes it hard to assess. Vaso-vagal episode day 2, night sweats, nausea and general fatigue and weakness	The most notable and hideously painful full back spasm episode. This type of spasm has the ability to completely immobilise me and is quite frightening to be honest. I need to remember to rise slowly from the lying down position to standing. I continue to experience the lower limb lateral pain in the right leg which I experienced pre-operatively, but there is marked	Still having some degree of lower lateral right leg pain and a very odd 4th right toe pain. Similar to the dorsal skin soreness experienced early on. I don't think it is the anti embolism stockings as there is no redness, a superficial soreness all over the 4th toe for something so small is fairly sore. After standing for a long period at the presentation	As above- fatigue. In addition when mobilising over longer distance a bit foggy. Had a bit of a shock when I gently shook my head after a shower. I got a jolt of pain that reverberated down my spine which was very unpleasant. I won't be doing that again. Still	General feeling of pain	Pain	Pain	Pain<br>Sleep	Pain	Pain<br>Sleep

Participant: P5	Entry					Categories/Themes				
**Prompt**	**Week 1**	**Week 2**	**Week 3**	**Week 4**	**Summary**	**Week 1**	**Week 2**	**Week 3**	**Week 4**	**Combined themes**
		improvement in the groin, knee and dorsal foot. I experienced some temporary slight numbness on the 2nd toe on the right foot which is now resolved, but had an odd almost sunburn like skin sensation over the right dorsal foot, there was no skin redness nor did I have a temperature, but not pleasant when covered by the anti embolism stockings and bedclothes etc. This seems also to be resolving. Some dull surgical site pain also being experienced but I very much expected this. The most overwhelming symptom this week I have experienced has probably been	I went to, I experienced the most horrid heavy leg pain bilaterally, posteriorally on both legs. This was a heavy feeling and my legs were restless all through the night. I therefore had a broken sleep and paid for it the next day.	have this weird 'sore 4th toe with some intermittent dorsal foot soreness' and the lower lateral right leg/ knee intermittent dull aching.						

**Participant: P5**	**Entry**					**Categories/Themes**				
**Prompt**	**Week 1**	**Week 2**	**Week 3**	**Week 4**	**Summary**	**Week 1**	**Week 2**	**Week 3**	**Week 4**	**Combined themes**
		the general fatigue and weakness. I guess my body is working hard to repair itself after the surgery, as well as the effects of the medication								
Medications you have taken this week	Tramadol, Paracetamol, Sumatriptan. Other narcotic analgesics, I/V antibiotics and anti-emetics given whilst in HDU. Senna, Lactulose	Tramadol, paracetamol, Senna, Lactulose.	Mainly Tramadol. Small amount of paracetamol. Trying hard to reduce the Tramadol.	Decided to get off the Tramadol. Very unpleasant side effects resulted in intense abdominal cramping with some nausea and diarrhoea which seems to be improving now and I'm still taking paracetamol	As and when needed dependent on pain threshold	Adherence	Manage	Manage	Manage	Adherence Management
Healthcare appointments this week	Nil	Nil	Wound check. Apparently the wound site has/is healing nicely.	Nil	Attendance	No data	No data	Access	No data	Access
Rehabilitation progress this week<br>- *Your ability to exercise*	Able to mobilise unaided for short walks. Able to ascend	Able to check work emails, write a reference for an employee,	Physically trying to extend the walks, able to do more	Sorting cupboards, cooking, cleaning,	Restricted mobility, increased confidence	Movement	Movement	Positive (+) Confident	Energy Positive	Energy Positive Movement

**Participant: P5**	**Entry**					**Categories/Themes**				
**Prompt**	**Week 1**	**Week 2**	**Week 3**	**Week 4**	**Summary**	**Week 1**	**Week 2**	**Week 3**	**Week 4**	**Combined themes**
and do activity this week *Return to work / activities / hobbies as relevant to you*	and descend stairs. Minimal assistance required with washing. Unable to complete any physical work but able to check and respond the work emails etc	watch TV, read, organise drawers, help keep the house tidy (with the assistance of my grabber). . Gentle mobilisation within personal limits. Enjoying catching up with visitors	household tasks and feeling I am contributing to the household. More confident with getting at lower level with good kneeling techniques to do extra household duties.	many more household duties and still doing some work from home						
Positive thought(s) this week	The surgery has now been completed. Delighted that it is over. We are hopefully going to reacquire the ability to walk for long periods without agonising pain. My family and friends- I am blessed and thankful for them and my faith in God has also helped me this week	I am definitely improving with regard to mobilising and other daily activities. Nerves are slow to heal, but they can heal.	Time is passing very quickly. Since discharge I have only had 1 day where I haven't had any visitors. I am very blessed and grateful. And I need to get better to show my friends and family that their support has assisted me with the process	Very positive but remaining realistic. So much depends on the complete success of the operation and so far I'm very pleased and thankful	Family support, realism in achievement	Support	Progress	Support	Positive (+)	Support Positive
Negative thought(s) this week	Fears of reoccurrence of pre-surgery symptoms. Am I going to be able to do what I did before?	Concerns with regard to the lower lateral discomfort in the right leg	Is that lower lateral right leg pain / discomfort ever going to completely go so I can get on	Will this lower right lateral leg pain and sore toe ever go? Will I be able to	Adequate recovery	Fear	Pain	Hopeful	Fear Anxiety	Fear Hope Anxiety

Participant: P5	Entry					Categories/Themes				
**Prompt**	**Week 1**	**Week 2**	**Week 3**	**Week 4**	**Summary**	**Week 1**	**Week 2**	**Week 3**	**Week 4**	**Combined themes**
			with my recovery? I'm aiming for 100% recovery, but I will be grateful for anything that allows me to walk/ exercise like I used to	run again... What if unusual exercise affects my surgery. When will the vertebrae and bony fusion be solid and strong?						
Most significant/memorable moment this week (may be positive or negative)	Seeing my family when I woke up in HDU. Appreciating the immediate benefit of pain relief was my most memorable moment. The realisation that a different type of pain / discomfort would need to be dealt with post operatively in order to continue making progress. Balancing pain medication relief is significant as it	Full back/ torso spasm (if that's what it was). I couldn't move or get comfortable fora few minutes which was very scary. The pain reduced me to tears, but this could have been coupled with anxiety. Luckily my husband was around- not that he could help in any way- it had to pass itself.	Going to my award ceremony in at the Council House with the Lord Mayor and also meeting with my researcher, as it was therapeutic talking about how my symptoms resulted in a surgical intervention and how that surgery now has the power to return me to my former health	Increasing independence- feeling I am contributing to the household / family activities again	Family support, encourage recovery	Support	Pain	Therapeutic	Energy	Energy<br>Pain<br>Therapeutic

Participant: P5	Entry					Categories/Themes				
**Prompt**	**Week 1**	**Week 2**	**Week 3**	**Week 4**	**Summary**	**Week 1**	**Week 2**	**Week 3**	**Week 4**	**Combined themes**
	impacts on everything									
Please describe how this week has been compared to last week	I'm obviously less mobile. I was at my full time job last week and a busy mum. This week I have had to submit to being cared for by my Drs, Nurses, Physio and family and friends. Having to rely on others when I am normally fiercely independent has been tough.	Definite improvement. Managing to reduce analgesia slightly. I do feel that I have 'turned a corner' with regard to my progress. Hopefully the only way is up from here on..	Improving, definite improvement generally physically and mentally	Improved.	Emotionally drained	Depression	Positive (+)	Progress	Positive (+)	Positive Depression
Please record any other comments you may like to make regarding how you have felt or your experiences over the past week?	I have moved from euphoria to feeling very low. Whether it is because I am also menopausal is difficult to know, but ultimately grateful to my surgeons and looking forward to getting on with working hard to get a good recovery and the best	Again thankful for the skill of the surgeons and the care of all allied health professionals. Overall this 2nd week has had both highs and lows	I know I must remain patient and not 'run before I can walk'. I know that nerves can take months to heal. There is definite improvement as my pre-operative groin and knee and dorsal right foot pain has virtually disappeared. I	Some frustrations with matters at work, but I must remember I am still on sick leave and I need to keep focused on my recovery. That must be priority. The wound site had	Staying hopeful, focussed on recovery	Positive (+)	Energy	Positive (+)	Frustration	Positive Energy

Participant: P5	Entry					Categories/Themes				
**Prompt**	**Week 1**	**Week 2**	**Week 3**	**Week 4**	**Summary**	**Week 1**	**Week 2**	**Week 3**	**Week 4**	**Combined themes**
	possible outcome		must stay positive and be hopeful that the intermittent lower right lateral leg pain will eventually improve. Again so grateful for the skills of the medical team.	been a bit swollen one side but seems to be resolving now						

Diary entry: P6

Participant: P6	Entry					Categories/Themes				
**Prompt**	**Week 1**	**Week 2**	**Week 3**	**Week 4**	**Summary**	**Week 1**	**Week 2**	**Week 3**	**Week 4**	**Combined themes**
Feelings about your progress this week	Slow progress being made	Feeling progress is a little better this week but a long way to go	Progress slowly gathering momentum	Going for walks of 30 mins showing good progress now able to sit longer	Recognising that recovery may be lengthy	Anxiety	Positive (+)	Speed	Positive (+)	Speed<br>Positive<br>Anxiety
Feelings about your recovery so far	Feeling quite weak suffering loose bowel movements	Recovering is making slow progress though I am able to do more things and exercise	All positive now walking upright	Quite happy as now I think I have unearthed causes of feet problem due to fall last year	Slow progress but remain hopeful	Fear	Speed	Positive (+)	Positive	Positive<br>Speed
Level of motivation	Feeling motivated but being patient as it will improve	I still have self motivation and even more after	Motivation good. Am able to drive again and use the hot tub	Have good motivation to get back to as normal as possible	Reasonable level of motivation	Hope	Hope	Positive	Hope	Hope<br>Positive

Participant: P6	Entry					Categories/Themes				
**Prompt**	**Week 1**	**Week 2**	**Week 3**	**Week 4**	**Summary**	**Week 1**	**Week 2**	**Week 3**	**Week 4**	**Combined themes**
		talking to surgeon								
Symptoms you have experienced over the past week	Loose bowel movements dehydration	Pullinh and slight pain in oeration area. Maybe due to late physio/scar tissue	Still minor pulling on back and buttocks	Minor twinges in back and buttocks	Pain at the wound site, irregular bowel movement	Movement	Pain	Pain	Pain	Pain Movement
Medications you have taken this week	Pain killers senna, dioralyte	Folic acid prescribed by local doctor pain killers but only when absolutely required omeprezole	Folic acid daily, painkillers as and when omeprezole	Folic acid daily less pain killers omeprezole	Managing medication according to pain threshold	Management	Management	Awareness	Management	Management Awareness
Healthcare appointments this week	In hospital overnight due to dehydration	Beginning of the week saw surgeon saw local doctor for results of blood test	Doctors to discuss effects of folic acid	First visit to physio	Access to healthcare	Access	Access	Access	Access	Access
Rehabilitation progress this week<br>- *Your ability to exercise and do activity this week*<br>*Return to work / activities / hobbies as relevant to you*	Little exercise done homebased	Able to do exercises and slowly increasing activities did Xmas decorations on tree	Able to carry out exercises and activities easier tidied out hypes and did a little cooking. Not yet back to hobbies	No problem with exercises and activities back to sewing short spells	Progressive with the ability to do some exercise	Speed	Movement	Positive (+)	Movement Positive (+)	Movement Positive
Positive thought(s) this week	Not much	Not a great deal of positives except being able to use Jacuzzi	Horay for my independence with driving and able to visit friends	Always positive and notice improvement	Adjusting to normal life, some positive	Speed	Positive (-)	Happy	Positive Energy	Positive Energy Speed

Participant: P6	Entry					Categories/Themes				
**Prompt**	**Week 1**	**Week 2**	**Week 3**	**Week 4**	**Summary**	**Week 1**	**Week 2**	**Week 3**	**Week 4**	**Combined themes**
Negative thought(s) this week	Feeling a little sorry for myself	Slow progress and still with slight pain very tired	None at moment	None really	Emotionally drained due to slow progress	Mood	Pain Anxiety	Positive (+)	Positive (+)	Positive Mood Anxiety
Most significant/memorable moment this week (may be positive or negative)	None	Being given the clearance to use hot tub	Driving and hot tub	Visit of granddaughter to cathedral to see trees xmas and just to see me	Good spirit due to support and reassurance	No data	Positive (+)	Mood	Support Mood	Mood Support
Please describe how this week has been compared to last week	Little improvement	No data	Progress is more pronounced	Each week is getting better	Slow progress but showing improvement	Slow	No data	Speed	Movement	Slow Movement Speed
Please record any other comments you may like to make regarding how you have felt or your experiences over the past week?	No data	Sleeping difficulty hence always tired	Still sleeping problems	Still sleeping problems	Sleep deprivation	Sleep Tiredness	Sleep	Sleep	Sleep	Sleep Tiredness

Diary entry: P7

Participant: P7	Entry					Categories/Themes				
**Prompt**	**Week 1**	**Week 2**	**Week 3**	**Week 4**	**Summary**	**Week 1**	**Week 2**	**Week 3**	**Week 4**	**Combined themes**
Feelings about your progress this week	Very positive about the way I feel real good	I really feeling some pain. Painful whenever I lay down or try to get up back to do anything	When I wake up in the morning I still feeling pain in my lower back	Movement are much better. I still feeling a little pain inside my back	Positivity however slight discomfort	Positive (+)	Pain	Pain	Movement	Positive Pain Movement
Feelings about your recovery so far	I'm better than before I know that I'm getting there	It really slow at this moment but I'm doing a lot of walking	Has long as I do my exercise and walk thing will be ok	Step by step. Better everyday	Steady recovery, positive attitude	Positive (+)	Energy	Energy	Positive (+)	Positive Energy

Participant: P7	Entry					Categories/Themes				
**Prompt**	**Week 1**	**Week 2**	**Week 3**	**Week 4**	**Summary**	**Week 1**	**Week 2**	**Week 3**	**Week 4**	**Combined themes**
Level of motivation	Feeling good	I'm feeling good with my legs each day as I go on	Everything I feel good within myself	Trying to do as much as possible. Is good	Feeling better and well-motivated	Positive (+)	Movement	Mood	Energy	Energy<br>Movement<br>Positive
Symptoms you have experienced over the past week	None	None	None	None	No data	No data	No data	No data	No data	No data
Medications you have taken this week	None	None	None	None	No data	No data	No data	No data	No data	No data
Healthcare appointments this week	It has been good news – 12/6/18 – 14/6/18	No	No data	None	Acceptable access to healthcare	Access	No data	No data	No data	Access
Rehabilitation progress this week<br>- *Your ability to exercise and do activity this week*<br>*Return to work / activities / hobbies as relevant to you*	Yes<br>Yes	I do my own exercise every day. I try to	Yes<br>yes	Fairly good<br><br>Not yet	Acceptable progress	Positive (+)	Motivation	Energy	Energy	Energy<br>Positive<br>Motivation
Positive thought(s) this week	I try to do things that I feel that can help ne improve myself	Good	I try to do more but it hurts if only I could do more	I'm happy so far to see how far I have come. Good	Self-motivation and driven	Positive (+)	Positive (+)	Pain	Positive (+)	Positive<br>Pain
Negative thought(s) this week	None	None	None	None	No data	No data	No data	No data	No data	No data
Most significant/memorable moment this week (may be positive or negative)	Having fun exercising everyday	I really don't have any but I try to be positive	More positive to do things	When I'm trying to do more walking	General feel good factor	Positive (+)	Positive	Positive	Energy	Positive<br>Energy
Please describe how this week has been compared to last week	Each week I feel better than I do	Not bad 7/10	I push myself to do more because I want	Better than before and	Gradual improvement. Feeling better	Energy	Energy	Energy	Positive (+)	Energy<br>Positive

Participant: P7	Entry					Categories/Themes				
**Prompt**	**Week 1**	**Week 2**	**Week 3**	**Week 4**	**Summary**	**Week 1**	**Week 2**	**Week 3**	**Week 4**	**Combined themes**
	expect so my feel or good		to see how far I go. Try to it better	I'm happy. good						
Please record any other comments you may like to make regarding how you have felt or your experiences over the past week?	There are sometime I feel pain but I don't make it stop me do anything it's not easy but I have to keep it up no matter what	Just whenever I try to get up when I lay down at any time after walking or exercise	My experiences was great all feel well within my motivation of doing things and getting things done so that make me feel good	Everything that I have done I'm so please with my progress throughout this week so far.	Self-motivation, steady progress	Pain	Movement	Energy	Positive (+)	Pain Positive Energy

Diary entry: P8

Participant: P8	Entry					Categories/Themes				
**Prompt**	**Week 1**	**Week 2**	**Week 3**	**Week 4**	**Summary**	**Week 1**	**Week 2**	**Week 3**	**Week 4**	**Combined themes**
Feelings about your progress this week	While in hospital I felt great On my return home I feel a little the disappointed	Up and down	Good	Great	Generally good, but apprehensive coming home	Anxiety	Positive (+)	Positive (+)	Positive (+)	Anxiety Positive
Feelings about your recovery so far	I feel optimistic no more sciatic pain bur still very sore	Good	Good	Positive	Feeling optimistic and generally positive	Positive (+)	Positive (+)	Positive (+)	Positive (+)	Positive
Level of motivation	High	High	High	Very High	Highly motivated	Driven	Driven	Driven	Driven	Driven
Symptoms you have experienced over the past week	Sharp Pain in my left side and buttock Bladder weakness and constipation	Pain getting up and down in left buttock	Pain in my pelvis and right knee	Pain in my left knee and calf muscles	Sustained pain associated with surgery and other symptoms	Pain	Pain	Pain	Pain	Pain

Participant: P8	Entry					Categories/Themes				
**Prompt**	**Week 1**	**Week 2**	**Week 3**	**Week 4**	**Summary**	**Week 1**	**Week 2**	**Week 3**	**Week 4**	**Combined themes**
					linked to medication					
Medications you have taken this week	2xdaily tramadol100ml slow release 2x150ml daily pregabalin 4x 50ml tramadol daily	2xdaily tramadol100ml slow release 2x150ml daily pregabalin 4x 50ml tramadol daily	Tramadol 100ml (both 2x daily tramadol 50ml	A few tramadol and ibuprofen	Awareness of how to manage medication	Adherence Awareness	Adherence	Adherence	Adherence	Adherence
Healthcare appointments this week	None	Nurse and Physio	Physio and nurse at practice	None	Access to healthcare	No data available	Access	Access	Access	Access
Rehabilitation progress this week<br>- *Your ability to exercise and do activity this week*<br>*Return to work / activities / hobbies as relevant to you*	I have walked a little with the Physio team I've exercised as shown in my post op guide I've been given none of the second question	As directed from hospital	I've been told to walk. I started with high goals. Which I have reduced a little	I have walked 30 minutes each day tried to walk uphill to build up muscles and fitness	Sporadic follow-up and unstructured care	Access	Access	Directed	Energy Goal	Energy Goal Access
Positive thought(s) this week	This pain will get better day by day	Had a big poo	Stitches out and the wound is almost. Healed so no dressing. Needed	My back pain is beginning to be a memory	Pathway to recovery under control	Positive (+)	Positive (+)	Positive (+)	Energy	Energy Positive (+)
Negative thought(s) this week	It hurts and I feel unsupported by the hospital now I'm home (i do the physio teams helpline to call if needed) Think I could do with a few aids at home to help e.g. The toilet is	Had to wait for the above until a toilet raiser was fitted 2 weeks post op	I wish I had been informed I'd had a bone graft from my pelvis before my visit.to the Physio as I could not understand the pain I was getting in that area	None	Feeling unsupported by the system	Pain Unsupported	Unsupported	Information	No data available	Unsupported Information Pain

Participant: P8	Entry					Categories/Themes				
**Prompt**	**Week 1**	**Week 2**	**Week 3**	**Week 4**	**Summary**	**Week 1**	**Week 2**	**Week 3**	**Week 4**	**Combined themes**
	too low and painful to sit down on. Also once I've got sat in bed I haven't anything to pull myself to standing position Which is also pay full and stressful									
Most significant/memorable moment this week (may be positive or negative)	Walking for the first time without the sciatic pain Wonderful!!	Able to Use the toilet	Walking home from the hospital.3 miles took me 2 hours but felt great	My birthday bbq ...I danced and felt full of fun and energy	Pain free and on tract to recovery	Energy<br>Positive (+)	Positive (+)<br>Energy	Positive (+)<br>Mood	Energy<br>Positive (+)	Positive<br>Mood<br>Energy
Please describe how this week has been compared to last week	I feel relief that I've finally had the op and it’s a fresh start	Every day I feel better than the last	I've been able to do more and building.my muscles Back up	I feel as though I am pain-free for the first time in 7 months	Improving and pain free	Positive (+)	Energy	Energy	Pain<br>Hopeful	Energy<br>Positive (+)<br>Hopeful
Please record any other comments you may like to make regarding how you have felt or your experiences over the past week?	Staff have all been great ..food all nice	I hope you don't get 5 of this	Enjoyed a bath and using public transport has been fun	I have had so many compliments from friends and family who haven't seen me post op..10 years have been taken off my face.	Grateful for the support received	Thankful	Mood	Energy<br>Positive (+)	Thankful	Thankful<br>Energy<br>Mood<br>Positive

Diary entry: P9

Participant: P9	Entry					Categories/Themes				
**Prompt**	**Week 1**	**Week 2**	**Week 3**	**Week 4**	**Summary**	**Week 1**	**Week 2**	**Week 3**	**Week 4**	**Combined themes**
Feelings about your progress this week	Don't feel that much progress has been made pain is still a major factor in being able to do much	Things are much the same as last week	Progress is slow process which can be hard to see only little change at a time	Have had both good and bad feelings earlier on in the week I was questioning myself if I had done the right thing , but this passed after a few days once my pain had eased a little , I think I had been pushing myself thinking about the time that had passed and how I was still feeling. During the last few days I have been taking things a little slower but still getting small tasks completed such as ironing. This	Slow progress, anxious and mentally drained	Anxiety<br>pain	Anxiety<br>Progress	Anxiety<br>Energy	Pain<br>Mood<br>Anxiety	Anxiety<br>Mood<br>Pain

Participant: P9	Entry					Categories/Themes				
**Prompt**	**Week 1**	**Week 2**	**Week 3**	**Week 4**	**Summary**	**Week 1**	**Week 2**	**Week 3**	**Week 4**	**Combined themes**
				made my feelings on my progress more positive,						
Feelings about your recovery so far	Pain is limiting my ability to move forward as much as I like	I have realised that its going to be a slow process	I feel that the recovery is slower than I thought it would be which can get you down some days	After speaking to family and friends I have come to see that my progress so far is positive	Acknowledgment that progress may be slow and progressive	Pain	Recognition	Anxiety Awareness	Positive (+) Support	Pain Recognition Anxiety Awareness Support
Level of motivation	Out of 10 level would be about 7	Out of 10, 7 again	Out of 10 : 6	Out of 10 : 6	Adequate level of motivation	Positive (+)	Positive (+)	Positive (+)	Positive (+)	Positive
Symptoms you have experienced over the past week	Pain in back pain in left leg only slight but feels much better	Back + leg pain weakness in legs continue	Back pain continues and depending on how much I have done I can also get burning down left leg into foot. At times left leg feels a bit weaker than the right	Still have lower back pain and pain in left leg and bottom that comes and goes depending on what I have been doing	General pain across back and leg	Pain	Pain	Pain	Pain	Pain
Medications you have taken this week	None	Gabapentine, paracetamol, zomorph, sertraline, lonzaprolol, epilim, mirabegren, diazepam, desmopressin	Gabapentin - 900mg Paracetamol Zomorph- 80mgs Sertraline 150mgs Lansprozol 15 mg Oxycodone as required	Gabapentin 900mgs Paracetamol Zomorph 80mgs Sertraline 150 MHz Lansprozol 15mgs Oxycodone as required	Prescribed and as required	Adherence Required	Adherence Required	Adherence Required	Adherence Required	Adherence Required

Participant: P9	Entry					Categories/Themes				
**Prompt**	**Week 1**	**Week 2**	**Week 3**	**Week 4**	**Summary**	**Week 1**	**Week 2**	**Week 3**	**Week 4**	**Combined themes**
Healthcare appointments this week	Practice nurse to remove dressing	None	None	None	Appears inconsistent	Access Limited	No data	No data	No data	No data
Rehabilitation progress this week<br>- *Your ability to exercise and do activity this week*<br>*Return to work / activities / hobbies as relevant to you*	Limited to pain<br><br>Limited due to pain and medication concentration	Stayed in mostly but was more active in daily chores<br><br>Not at work and hobbies I do still not able to concentrate	Not yet back at work, I am able to do light house work and my body tells me when I need to stop and rest	No back at work Am trying to do things as my pain allows I feel that I am able to do more this week if take things slow	Affected by pain and medication	Pain reduce	Mood	Movement	Pain Positive (+)	Mood Pain Positive
Positive thought(s) this week	Things can only get better I hope	Can't believe how time goes quickly	I am able to move start to do more little things around the house	That I can do things independently if I take it slowly having tests as required	Positive attitude and acknowledgement that progress is going to be progressive and slow	Positive (+)	Positive (+)	Movement	Energy	Positive Movement Energy
Negative thought(s) this week	Confusion to why leg feels funny/weak	Had a few days regretting having the operation due to pain	Thought that the recovery would be quicker and not as painful	I want to be able to do more things like driving, but have been told to wait 6 weeks, and I don't feel ready yet anyway and is gets to me	Realisation that recovering is going to be slow and progressive	Anxiety	Pain	Anxiety Mood	Frustration	Anxiety Mood Frustration
Most significant/memorable moment this week (may be positive or negative)	Returning home from hospital and being able to do small tasks	The regret for having the operation done	I am slowly starting to see little improvements	Talking to friends and family more about their thoughts on my progress so far, and getting	Realisation recovery following surgery is mentally draining and frustrating at times	Positive (+)	Anxiety Regret	Positive	Support	Positive Anxiety Regret

Participant: P9	Entry					Categories/Themes				
Prompt	Week 1	Week 2	Week 3	Week 4	Summary	Week 1	Week 2	Week 3	Week 4	Combined themes
				positive answers back , this lifted my mood and made me think that I am to hard on myself sometimes						
Please describe how this week has been compared to last week	Was in hospital last week	Today I am slightly positive that all will go well and it just takes time	I can do more housework and small improvements can be seen	This week I have been able to do more things if I take my time , I have also realised more that I am making slow progress						
Please record any other comments you may like to make regarding how you have felt or your experiences over the past week?	None	None	None	None	No data	No data	No data	No data	No data	No data

Diary entry: P10

Participant: P10	Entry					Categories/Themes				
**Prompt**	**Week 1**	**Week 2**	**Week 3**	**Week 4**	**Summary**	**Week 1**	**Week 2**	**Week 3**	**Week 4**	**Combined themes**
Feelings about your progress this week	Very positive, awoke in recovery room immediately noticing that the underlying neuropathic pain down the back of my left leg had disappeared. First two days at home experienced mild to moderate pain around the base of my spine, but nothing excessive.	Not as good as the previous week, severe pain reaching a climax on Wednesday afternoon when the only position that I could get any degree of comfort was lying face down on the bed with a pillow supporting my stomach. Fortunately the pain started to recede from then on.	Much better than previous week, although still feeling original pain down the back of my left leg.	No progress from last week, still feeling original pain down the back of my left leg.	Pathway to recovery appears to be slow and steady with period of severe pain	Positive (+) Hopeful	Pain	Positive (+)	Pain	Pain Positive Hopeful
Feelings about your recovery so far	I felt better than I had expected to at this point.	Not so good, disappointingly the original pain down the back of my left leg started again on Thursday, not sure whether this is normal so a bit worried.	Still worried that the procedure may not have been a success as still having pain described above. Obtained an earlier appointment with my consultant	Still worried that the procedure may not have been a success as still having pain described above.	Mixed feeling about procedure and recovery	Positive (+)	Anxiety	Anxiety	Anxiety Pain	Positive Anxiety Pain
Level of motivation	Very good, able to walk around the apartment with the aid of	Still good, able to walk around the apartment with the aid of	As last week still good, able to walk around the apartment	As last week still good, able to walk around the	Improved ability to walk	Movement	Positive (+)	Positive (+)	Pain	Positive Pain

Participant: P10	Entry					Categories/Themes				
**Prompt**	**Week 1**	**Week 2**	**Week 3**	**Week 4**	**Summary**	**Week 1**	**Week 2**	**Week 3**	**Week 4**	**Combined themes**
	crutches at frequent intervals.	one crutch at frequent intervals. Can't stay on my feet for more than 10 minutes or so due to pain building up down the back of my left leg.	with the aid of one crutch at frequent intervals. Can't stay on my feet for more than 10 minutes or so due to pain building up down the back of my left leg. Can walk without the aid of a crutch, but still using one in case of unsteadiness.	apartment with the aid of one crutch at frequent intervals. Can't stay on my feet for more than 10 minutes or so due to pain building up down the back of my left leg. Can walk without the aid of a crutch, but still using one in case of unsteadiness.						
Symptoms you have experienced over the past week	Mid to moderate pain around the site of the operation.	Severe to medium pain around the site of the operation. Painkillers appear to be keeping this at bay by the end of the week.	Mild pain around the site of the operation. Painkillers appear to be keeping this at bay	No pain around the site of the operation. Wound totally healed now, painless when touched. Amount of time that I can stay on my feet before recurrence of pain down the back of my left leg varies from day to day, would say 80/20 in favour of bad	Varying degree of pain throughout especially around wound site	Pain	Pain	Pain	Positive (+)	Positive Pain

Participant: P10	Entry					Categories/Themes				
**Prompt**	**Week 1**	**Week 2**	**Week 3**	**Week 4**	**Summary**	**Week 1**	**Week 2**	**Week 3**	**Week 4**	**Combined themes**
				days. Wednesday and Sunday were good days this week.						
Medications you have taken this week	Amitriptylene, Toltoredine. Pregablin, Co-codamol and Oromorph	Amitriptylene, Toltoredine. Pregablin, Co-codamol and Oromorph	Amitriptylene, Toltoredine. Pregablin, Co-codamol and Oromorph	Amitriptylene, Toltoredine. Pregablin, Co-codamol and Oramorph	As prescribed	Adherence Awareness	Adherence Awareness	Adherence Awareness	Adherence Awareness	Adherence Awareness
Healthcare appointments this week	none	District Nurse visited on Friday, says the wound is healing well with no sign of infection.	District Nurse visited on Wednesday, says the wound is healing well with no sign of infection. Wound not requiring a dressing now. Signed off, no more visits planned.	None	Access to health care professionals	No data	Access	Access	No data	Access
Rehabilitation progress this week<br>- *Your ability to exercise and do activity this week*<br>*Return to work / activities / hobbies as relevant to you*	Able to walk with the aid of crutches. Nothing else at this point.	Able to walk with the aid of one crutch. Doing exercises three times a day from Thursday onwards. Picked my guitar up a few times and played for five minutes or so during the latter part of the week.	Able to walk with the aid of one crutch. Doing exercises three times a day. Played guitar a few times.	Able to walk with the aid of one crutch. Doing exercises three times a day. Played guitar a few times.	Ability to walk with crutches	Movement	Movement	Movement	Movement	Movement
Positive thought(s) this week		None	Able to walk for longer periods.	No problems with infection.						

Participant: P10	Entry					Categories/Themes				
**Prompt**	**Week 1**	**Week 2**	**Week 3**	**Week 4**	**Summary**	**Week 1**	**Week 2**	**Week 3**	**Week 4**	**Combined themes**
	Feeling optimistic about the outcome. Less pain than I expected.		No problems with infection.	Wound completely healed.						
Negative thought(s) this week	None	Feeling a bit down due to re-occurrence of original pain.	As last week still feeling a bit down due to re-occurrence of original pain.	As last week still feeling a bit down due to re-occurrence of original pain.	Psychologically drained due to recurring pain	No data	Depression	Depression	Anxiety	Anxiety Depression
Most significant/memorable moment this week (may be positive or negative)	Being discharged from hospital.	Re-occurrence of original pain.	Re-occurrence of original pain still most significant problem.	Re-occurrence of original pain still most significant problem.	Recurring pain	Positive (+)	Pain	Pain	Pain	Pain
Please describe how this week has been compared to last week	N/A First week.	Much more painful than the previous week but subsided by the end of the week.	Post-operative pain has receded from the previous week's level.	No post-operative pain now.	Pain under control	No data	Pain	Recede	Energy	Pain Recede Energy
Please record any other comments you may like to make regarding how you have felt or your experiences over the past week?	Discharged from Hospital so not much to report this week.	The first few days after the operation were so good and free of severe pain that this week came as a big shock, I hadn't realized how painful it would be	None.	None.	Managing pain following surgery	Positive (+)	Pain	No data	No data	Positive Pain

Diary entry: P11

Participant: P11	Entry					Categories/Themes				
**Prompt**	**Week 1**	**Week 2**	**Week 3**	**Week 4**	**Summary**	**Week 1**	**Week 2**	**Week 3**	**Week 4**	**Combined themes**
Feelings about your progress this week	Slow but nice to be home	Slow but then I realised I could open a drawer which I couldn't do a week ago	Slow until I look bad and have achieved. No night light reduction in codeine	Slow - but I'm probably impatient	Slow progress	Energy	Energy	Positive (+)	Frustration	Frustration Energy Positive
Feelings about your recovery so far	Very slow	Still got a long way to go	Slow	Again slow - but as for last week until I look back. Achieved getting in my special sports car and a couple of run outs in it along with joining club members, who I've never met before, for breakfast. Still no desire to drive (or have any alcohol!!)	Slow recovery but hopeful with support from friends	Frustration	Speed	Speed	Speed Support	Speed Support
Level of motivation	Got to carry on	Slow and steady do what the body wants	Must carry on I have no desire to remain incapacitated and rely on others	I will continue to improve.	Slow recovery but desire to improve	Mood	Energy	Mood	Positive (+)	Mood Energy Positive
Symptoms you have experienced over the past week	Very limited bowel movement discomfort on peeing	Continued discomfort sore right calf lavatories problems continue	Waking roughly every two hours in the night to wee	Reduction of pain. But 'night cramps' in right leg continue to make the	Symptoms include irregular bowel movement,	Movement	Pain	Sleep	Sleep Pain	Movement Pain Sleep

Participant: P11	Entry					Categories/Themes				
**Prompt**	**Week 1**	**Week 2**	**Week 3**	**Week 4**	**Summary**	**Week 1**	**Week 2**	**Week 3**	**Week 4**	**Combined themes**
				stairs difficult (especially going down). Bowels can be reluctant to open and generally roughly every two hours I need a wee during the night, so sleep remains broken	disturb sleep pattern					
Medications you have taken this week	Codeine	Reducing codeine slowly	Paracetamol reducing codeine e.g. 2 on Saturday, I on Sunday, movoicol	Stopped the dihydracodein. Only one or two paracetamol taken for bed. But taking supplements - fish oil as it can reduce inflammation and pills to increase gut bacteria which generally helps everything and zinc and copper - can't remember what the nutrionist says that helps, but I have seen it confirmed on the internet.	As prescribed	Adherence Awareness	Adherence Awareness	Adherence Awareness	Adherence Awareness	Adherence Awareness

Participant: P11	Entry					Categories/Themes				
**Prompt**	**Week 1**	**Week 2**	**Week 3**	**Week 4**	**Summary**	**Week 1**	**Week 2**	**Week 3**	**Week 4**	**Combined themes**
Healthcare appointments this week	Released from hospital on Thursday	Had clips removed from a lot larger than expected incision	None	None - although your researcher came to interview me and told me that my metal work is quite extensive - I had no idea and am looking forward, hopefully, to seeing the X-ray when I see the surgeon at the beginning of November	Healthcare access adequate	Positive (+)	Access	No data	Access	Access Positive
Rehabilitation progress this week<br>- *Your ability to exercise and do activity this week*<br>*Return to work / activities / hobbies as relevant to you*	All very limited just wait and managed stairs slowly	Aware that right leg is quite weak so concentrating on it on the stairs	Continue and extend walking do more chores around house e.g. laundry	Walking has improved - both in term of distance and speed, and, according to my neighbours, it no longer appears I am waring a large nappy!<br>No thoughts on returning to work or hobbies	Making reasonable progress	Energy	Awareness	Energy	Speed Energy	Energy Awareness Speed
Positive thought(s) this week	None	1/3 away through of coping with aeroplane socks	Nice to go out	Fantastic to be out in my beloved car	Managing recovery	No data	Positive (+)	Mood	Mood	Mood Positive

Participant: P11	Entry					Categories/Themes				
**Prompt**	**Week 1**	**Week 2**	**Week 3**	**Week 4**	**Summary**	**Week 1**	**Week 2**	**Week 3**	**Week 4**	**Combined themes**
Negative thought(s) this week	None	none	I hate being cold I got rather chilly on Saturday evening possibly did too much	I don't like being trapped beholden on others to take me out	Unwilling to accept assistance	No data	No data	Energy	Mood Frustration	Mood Frustration Energy
Most significant/memorable moment this week (may be positive or negative)	Getting out of hospital	Made soup and at in front of computer	Going out for tea	Going out in my beloved car - with the extra bonus was that Saturday was a lovely sunny day	Feeling independent	Mood	Positive (+)	Energy	Energy Mood	Mood Energy Positive
Please describe how this week has been compared to last week	No data	Unaware of progress until think about it e.g. easier to access microwave	Can do more when looking for company. The same	Further improvement when I look back, although I cannot really do any more tasks (all I really do is my own personal hygiene, the washing up and the laundry), those that I do I do quicker.	Good progress	No data	Positive (+)	Positive (+)	Speed	Speed Positive
Please record any other comments you may like to make regarding how you have felt or your	No data	No data	More achievement. Can put on and take off compression stocking myself	No data	Good progress and self-motivated	No data	No data	Self-efficacy	No data	Self-efficacy

Participant: P11	Entry					Categories/Themes				
Prompt	Week 1	Week 2	Week 3	Week 4	Summary	Week 1	Week 2	Week 3	Week 4	Combined themes
experiences over the past week?			so can have a shower whenever I want. Can load/unload hang out the washing without help. Wanted to be driver to town (1 mile) to do some shopping. Went to a farm shop for tea and had a nice drive round country side							

Diary entry: P12

Participant: P12	Entry					Categories/Themes				
Prompt	Week 1	Week 2	Week 3	Week 4	Summary	Week 1	Week 2	Week 3	Week 4	Combined themes
Feelings about your progress this week	Feel like I have not progressed that much, stood up and walked to the bathroom	Managed to walk upstairs prior to leaving hospital. Apart from walking up and down stairs I have walked outside the front of the house	Managed to sleep in bed. My Mom+Dad has given me their bed bars to assist in getting out of bed so I am a lot better but not feeling too daring yet	I am able to walk better this week. Managed to shop but was too far a struggled to get back. Slept for 2 hours when I got back, had to take more pain relief	Steady pace and cautious of not overdoing	Anxiety	Positive (+)	Positive	Fatigue<br>Anxiety<br>Sleep	Anxiety<br>Positive<br>Fatigue<br>Sleep
Feelings about your recovery so far	Mentally pain and lack of sleep makes me feel I	Still only managing 1.5 hours sleep	Much better walking better and a bit further	Much better sleeping 3 hours up for	Slow and mentally draining.	Mood<br>Sleep	Sleep	Positive (+)	Sleep	Mood<br>Sleep<br>Positive

Participant: P12	Entry					Categories/Themes				
**Prompt**	**Week 1**	**Week 2**	**Week 3**	**Week 4**	**Summary**	**Week 1**	**Week 2**	**Week 3**	**Week 4**	**Combined themes**
	have not started to recover	before I wake up go to the toilet then 1.5 hours sleep. Sleeping on sofa		a toilet break, then 2 hours every night. Almost back to 7 hours a night	Improved sleep patter					
Level of motivation	No mention, I am tired of all pain and no sleep (-10)	Due to no sleep I feel a lack of motivation. This got no better this week – 10 turned into a 0	As I can sit up without pain and can play games with the kids I am feeling happier probably up to a 4/5 from previous 0 score	Motivation is ok. I want to get a routine going so I started to get up have breakfast and exercise then get showered light walk lunch sleep walk dinner bed for last 2 days of the week	Motivation affected by tiredness and pain.	Fatigue	Sleep	Positive (+)	Positive (+)	Positive<br>Sleep<br>Fatigue
Symptoms you have experienced over the past week	Pain around area that was operated on, goes down leg in left side	Still pain in back and legs now are on fire. Can't feel the other parts on the top of thighs stinging/burning sensation only	Back pain severe when I relax lying down, aches and pains are constant but shooting pain down my left leg occurs I tense up and give myself a headache. Leg just as sore	Lower back pain is avoided by moving in ways to avoid it. Taking lower dose of aramorph 3mls twice a day	Generalised pain around wound area	Pain	Pain	Pain	Pain	Pain
Medications you have taken this week	Zomorph<br>Oromorph<br>Tramadol	Zomorph<br>Oromorph<br>Senna	Zomorph<br>Oromorph<br>Senna	All still being taken, 3 mls from	As prescribed	Adherence	Adherence	Adherence	Adherence	Adherence

Participant: P12	Entry					Categories/Themes				
**Prompt**	**Week 1**	**Week 2**	**Week 3**	**Week 4**	**Summary**	**Week 1**	**Week 2**	**Week 3**	**Week 4**	**Combined themes**
	Paracetamol<br>Senna<br>Lactulose	Paracetamol<br>Osteocare vitamin<br>Lactulose	Paracetamol<br>Osteocare vitamin<br>Lactulose<br>arnica	5 mls of oromorph twice a day						
Healthcare appointments this week	No data	No data	Doctors for wound dressing to be removed booked blood tests for Monday	I had blood tests on Monday	Appear to have limited access to health care	No data	No data	Access	Access	Access
Rehabilitation progress this week<br>- *Your ability to exercise and do activity this week*<br>*Return to work / activities / hobbies as relevant to you*	Stood up day 1 Stood up and sat down and walked. Walked odd days. Sit down strengthen as I walked	Continue to walk out front of house. Tried a mobility electric chair to help me go up room. Sit down stretches as much as I can	Continuing to walk daily. Can't sit on floor yet to play with kids but close	Walked further but went too far. Not sure what I should be doing	Motivation to stay mobile to aid recovery	Movement	Movement	Movement	Movement<br>Frustration	Movement<br>Frustration
Positive thought(s) this week	Not had any.	Went to watch Layla swim, it was painful to sit but managed it	Managing to sleep in won bed with my wife is great. Recently purchased the bed for £1,600 so sleeping in it is<br>great	Thought about how I would be at work	Impact on psychological well-being	Anxiety<br>Mood	Positive (+)	Mood	Anxiety	Mood<br>Anxiety<br>Positive
Negative thought(s) this week	Feel like it hasn't worked feel like I understand OP stressed just before OP as they said I wasn't going to have it. Then ok	Level of sleep is really manage it difficult I am pretty miserable	Confused to what I should/shouldn't be doing this is pissing me off at times as boredom has crept in massively	Thought about how I would be at work	Cannot establish a proper plan of recovery	Anxiety	Sleep	Mood	Anxiety	Anxiety<br>Sleep<br>Mood

Participant: P12	Entry					Categories/Themes				
**Prompt**	**Week 1**	**Week 2**	**Week 3**	**Week 4**	**Summary**	**Week 1**	**Week 2**	**Week 3**	**Week 4**	**Combined themes**
Most significant/memorable moment this week (may be positive or negative)	I walked	Got outside being very snappy to kids	Managing 3 hours un interrupted sleep was positive	My stretching has allowed me to sit on floor to play with the kids	Psychologically challenging	Movement	Mood	Sleep	Positive (+)	Movement Mood Sleep Positive
Please describe how this week has been compared to last week	I could not compare	A bit more mobile. Not where I want to be/expected to be	Improved this week. To 40% to normal	Moved on 10% this week feel like I'm half way to recovery	Movement still restricted	Frustration	Movement	Positive (+)	Positive (+)	Movement Positive
Please record any other comments you may like to make regarding how you have felt or your experiences over the past week?	I severely underestimated this operation. I did not expect the operation to be 9 hours. I felt like I would be in pain but able to sleep and walk. I also was badly bruised on my head, chest, hip and feet. After being on the operating table this also hurts, is uncomfortable. Prior to the operation I was told I would need to quit smoking, to assist with recovery. So I slowed smoking	My level of expectation to walk I would be at able to walk was 60%/70% recovered. I am approximately half even less at 25%.	All operations, Dr at least big operations should come with a rough guide of what to expect on a weekly basis. Also the dos and dont's should be clear before being let out of hospital. I enjoyed my time in hospital as it gave me an insight to what nurses go through having only heard it from my wife but my discharge from hospital was like I was being kicked out. I didn't feel	I have concerns that my legs are not any different lump around my scar hurts when I lie on it. Where my drain was is sore and has gone into bum cheeks	Concern about the speed of recovery	Anxiety Frustration	Anxiety	Anxiety Frustration	Pain	Anxiety Frustration Pain

Participant: P12	Entry					Categories/Themes				
**Prompt**	**Week 1**	**Week 2**	**Week 3**	**Week 4**	**Summary**	**Week 1**	**Week 2**	**Week 3**	**Week 4**	**Combined themes**
	down and stopped prior to the operation. I was told on the day they would not do the operation. I was told on the day they would not do the operation due to me smoking still. I mix up in miscalculation almost cost me to not have the operation. If it was that important I would have quit when it was agreed to do the operation.		ready or clued up on what I had to when I left							

Diary entry: P13

Participant: P13	Entry					Categories/Themes				
**Prompt**	**Week 1**	**Week 2**	**Week 3**	**Week 4**	**Summary**	**Week 1**	**Week 2**	**Week 3**	**Week 4**	**Combined themes**
Feelings about your progress this week	Still having problems getting up from chair to standing	I feel I'm not making enough progress with regard to the back pain	Progress is very slow I need to sort my medication for back pain	I am not having pain down my left leg so feel the operation has done what it was supposed to do	Not enough progress has been achieved. Movement still restricted	Movement	Frustration	Pain Anxiety	Positive (+)	Movement Frustration Pain Anxiety

Participant: P13	Entry					Categories/Themes				
**Prompt**	**Week 1**	**Week 2**	**Week 3**	**Week 4**	**Summary**	**Week 1**	**Week 2**	**Week 3**	**Week 4**	**Combined themes**
Feelings about your recovery so far	I feel I need some physio	I haven't had pins and needles in my left leg as before the operation	I'm getting negative feelings about the pain	No data	Feeling that follow-up care is inadequate	Anxiety	Pain<br>Anxiety	Anxiety	No data	Anxiety<br>Pain
Level of motivation	Low	Medium to low motivation	Low	Low to medium	Motivation low throughout	Impetus (-)	Impetus (-)	Impetus (-)	Impetus (-)	Impetus
Symptoms you have experienced over the past week	I seem to be leaning forward	Pain in my lower back	Back pain is my biggest problem	Back pain is no easier	General complain of back pain	Movement	Pain	Pain	Pain	Movement<br>Pain
Medications you have taken this week	Aspiring 75mg daily, amitriptyline 10mg, 2xnight, adical 1500 mgx2 night, levothyroxine 100mg 1 daily vesicare 5mg 1 daily, Ramipril 1,25mg 1 daily, gabapentin 300mg 2x3 daily, arava 20mg 1 fdaily, bisoprolol 2.5mg 1 fdaily longtec 30mg, lansoprazole 30mg, atorvastatin 80mg	Same as before	As normal	Normal medication	As prescribed	Adherence<br>Awareness	Adherence<br>Awareness	Adherence<br>Awareness	Adherence<br>Awareness	Adherence<br>Awareness
Healthcare appointments this week	No data	Had the flu jab on the 10th Nov	I saw my consultant he arranged for x rays on my spine. The x-ray shows that everything was	Spoke with my consultant about my arthritis and how much this is to do	Reassurance from Surgeon, other issues discussed.	No data	Access	Access	Access	Access

Participant: P13	Entry					Categories/Themes				
**Prompt**	**Week 1**	**Week 2**	**Week 3**	**Week 4**	**Summary**	**Week 1**	**Week 2**	**Week 3**	**Week 4**	**Combined themes**
			normal regarding the operation	with the back pain						
Rehabilitation progress this week<br>- *Your ability to exercise and do activity this week*<br>*Return to work / activities / hobbies as relevant to you*	Still having pain on exercise	I am doing very little exercises due to back pain	No data	No data	Rehabilitation slow due to little activity	Pain	Anxiety	No data	No data	Pain<br>Anxiety
Positive thought(s) this week	Hoping to improve	Hoping to improve	My consultant said I was doing well and thought it was early days	Looking forward to Christmas with all the family	Support from Surgeon appears to have a positive impact	Positive (+)	Positive (+)	Support	Mood	Positive<br>Support
Negative thought(s) this week	Not getting any better	Still having problems with mobility	Still not improving enough	Condition not getting any better	Recovery appears to be longer	Anxiety	Movement	Anxiety	Anxiety	Movement<br>Anxiety
Most significant/memorable moment this week (may be positive or negative)	Having to keep have a rest when walking	I managed to go up my daughters stairs with a little help	I managed to go to the hairdresser I sat for 2½ hours	The pain in my lower back isn't improving	Pain is the main issue also related to level of energy	Fatigue	Movement	Movement	Pain	Fatigue<br>Pain<br>Movement
Please describe how this week has been compared to last week	Mush the same	Its still been the pain in the bottom of my back. Its shown no improvement	I am still struggling with pain the same	It's been about the same	Sustained pain throughout	Anxiety	Pain	Pain	Pain	Pain<br>Anxiety
Please record any other comments you may like to make regarding how you have felt or your experiences over the past week?	I have pain in my left lower back so I can't walk very far	Nothing seem to be changing	No data	The amount of pain I am still having is making my mood low	Mood and general psychological well-being affected by pain	Anxiety	Anxiety<br>Mood	No data	Mood	Mood<br>Anxiety

Diary entry: P14

Participant: P14	Entry					Categories/Themes				
**Prompt**	**Week 1**	**Week 2**	**Week 3**	**Week 4**	**Summary**	**Week 1**	**Week 2**	**Week 3**	**Week 4**	**Combined themes**
Feelings about your progress this week	It was a very traumatic week as left hospital sooner than I thought as I was in only one night. I was walking very well when I left but the day after I could hardly walk or move. The effort to pull myself up the stairs and sit down on the toilet. The progress over the week since then has been very good as I can walk up the stairs without having to pull myself up	The week has been very up and down, some days good, not sure how much I can do so having to be really careful as not to overdue it.	I feel that my progress has slowed up after the initial healing.	Slow	Mixed progress	Movement Anxiety Positive (+)	Movement	Movement Anxiety	Movement	Movement Anxiety Positive
Feelings about your recovery so far	My recovery this first week after the op has been very up and down with the release of the sciatic nerve throwing up different problems. I am moving more freely than at the beginning of the	Well I think that it's going alright at the moment but it's still early days.	It's felt like it's sort of levelled out	Still feels like it's not getting very far yet	Recovery very slow	Anxiety	Positive (+)	Positive(+)	Mood	Anxiety Positive Mood

Participant: P14	Entry					Categories/Themes				
**Prompt**	**Week 1**	**Week 2**	**Week 3**	**Week 4**	**Summary**	**Week 1**	**Week 2**	**Week 3**	**Week 4**	**Combined themes**
	week. Not able to sleep up in bed no more than two hours at night before I have to come down and sleep in the recliner for the rest of the night									
Level of motivation	I would like to be able to do some of the simplest things for myself instead of relying on my wife to do as she suffers from Ankylosing Spondylitis. The extra things that she is having to do is tiring her out tremendously. To be able to do things for myself will make me feel better instead of having to rely on someone else	To be able to get back to do normal things	It's still to be able to help the wife and take some pressure off her with doing household things	It's still to be able to do more chores around the house to help the wife.	Emotionally drained, being unable to contribute	Anxiety Frustration	Positive (+)	Helpful	Frustration	Positive Anxiety Frustration
Symptoms you have experienced over the past week	Extreme pain in the lower back where I had the op. Agony in the left leg from the Sciatic nerve from when it was released by the op. Pain has subsided in the leg a lot since the	Had a lot of pain in the left leg and back earlier in the week. Left leg and hip aches a lot. Have found that I can't sleep in the bed yet for more than around 2 hours so have given up trying	Aching across the lower back and left hip. Stiffening up if I sit for too long and don't get up and move. Wound is still healing and the scabs are reducing	Aching in the muscles across the lower back and buttocks. Left hip aching. Scar tissue stings occasionally. Lower back and hip	General pain	Pain	Pain	Pain	Pain	Pain

Participant: P14	Entry					Categories/Themes				
**Prompt**	**Week 1**	**Week 2**	**Week 3**	**Week 4**	**Summary**	**Week 1**	**Week 2**	**Week 3**	**Week 4**	**Combined themes**
	op. I've had a rash with peeling skin during the week which I had to have a home visit for. Had some side effects from the painkiller tablets I was prescribed from the hospital.	and sleep in the recliner chair. Had one night where I couldn't sleep. I do get tired a lot during the day.		stiffens up if I sit for too long						
Medications you have taken this week	No data	Had an appointment with a nurse at the doctors on Thursday to check that the stitches had dissolved	No data	No data	No medication review	No data	Review	No data	No data	Review
Healthcare appointments this week	Had to get the wife to take my notes into the Doctors for more painkillers as not enough given to cover the first week. Had to have a home visit from the doctors to check out a rash with peeling skin. A cream was prescribed.	Getting up and walking about more to exercise the leg and hip and muscles in the lower back	None	None	Some but inadequate advice	Frustration	Movement	No data	No data	Frustration Movement
Rehabilitation progress this week<br>- *Your ability to exercise and do activity this week*	Wasn't given any exercises to do so not sure what is safe or not to do. I'm just walking, stretching my legs. Was told	Getting up and walking about more to exercise the leg and hip and muscles in the lower back	Wasn't sure what I could do exercise or activity wise.	Standing and walking around more	Inadequate instruction and help	Anxiety Stress	Movement	Movement Frustration	Movement	Frustration Anxiety Stress Movement

**Participant: P14**	**Entry**					**Categories/Themes**				
**Prompt**	**Week 1**	**Week 2**	**Week 3**	**Week 4**	**Summary**	**Week 1**	**Week 2**	**Week 3**	**Week 4**	**Combined themes**
Return to work / activities / hobbies as relevant to you	not to lift anything heavy but not sure what is actually heavy and for how long I'm not supposed to not lift. I've been doing little bits of things to keep myself occupied but I'm not supposed to lift up my laptop so have to rely on my wife.									
Positive thought(s) this week	Not had any	Moving a little better	To achieve more by moving about	Went out the house twice this week on trips.	Speed up recovery and a return to normal activity	Positive (-)	Movement	Movement Positive (-)	Social	Social Movement Positive
Negative thought(s) this week	Why did I do it.	So unsure of what I can do safely	Frustrated on the limitation on what I can do and still not able to sleep in the bed.	The trips really tired me out.	Frustrated because of pain, discomfort and limitation due to surgery	Frustration	Anxiety	Frustration	Fatigue	Fatigue Frustration Anxiety
Most significant/memorable moment this week (may be positive or negative)	Able to get down and up off the toilet easily. Climbed the stairs and walked down them without struggling	Walking better	Had a friend visit	Had my birthday and went out for the day	Mobility improved and socialising	Movement	Movement Energy	Support	Social	Movement Support Social Energy
Please describe how this week has been compared to last week	Last week I hadn't had the op. This week very	Less pain from the actual operation	Just a slight improvement on my movement	A little improvement in movement,	Slow improvement in mobility	Pain Stress	Positive (+)	Anxiety	Movement Slow	Pain Positive Movement Anxiety

Participant: P14	Entry					Categories/Themes				
**Prompt**	**Week 1**	**Week 2**	**Week 3**	**Week 4**	**Summary**	**Week 1**	**Week 2**	**Week 3**	**Week 4**	**Combined themes**
	painful and stressful		but not much else.	recovery has seemed too slowed up.						
Please record any other comments you may like to make regarding how you have felt or your experiences over the past week?	Not enough information was forth coming about what aftercare would be needed or precisely what I could or could not do. The wife was getting stressed about that fact. I had to had a hand rail fitted to the toilet wall so that I could get up and down on the toilet. Felt very frustrated and down with the pain and the helplessness	Not felt so good in myself, a little down. Not liking seeing my wife having to struggle to do everything	Have felt very frustrated and low with not knowing my capability limits	It's been difficult to get a balance between sitting down and standing up and walking around. Too much of each can hurt. Kept falling asleep during the day a lot	Issues with follow-up healthcare	Inadequate	Anxiety Mood	Frustration	Mood Frustration	Mood Frustration Anxiety Inadequate

Diary entry: P15

Participant: P15	Entry					Categories/Themes				
**Prompt**	**Week 1**	**Week 2**	**Week 3**	**Week 4**	**Summary**	**Week 1**	**Week 2**	**Week 3**	**Week 4**	**Combined themes**
Feelings about your progress this week	Great progress now mostly clear of pre-existing leg pain	Progress continues slowly following last week's great gain	Progress continues slowly	No data	Steady progress	Movement	Movement Positive (+)	Movement Positive (+)	No data	Movement Positive
Feelings about your recovery so far	Wound healed (finally)	Happy but could do with more	Pleased with progress	No data	Reasonable progress with recovery	Movement Speed	Movement Positive (-)	Positive (-)	No data	Positive Movement Speed

Participant: P15	Entry					Categories/Themes				
**Prompt**	**Week 1**	**Week 2**	**Week 3**	**Week 4**	**Summary**	**Week 1**	**Week 2**	**Week 3**	**Week 4**	**Combined themes**
	Was hoping for more progress with pre-existing pain	guidance on exercise.								
Level of motivation	OK. Wish I knew what exercise to take. I am frightened to do much without guidance	OK. Wish I knew what exercise to take. Seeing Consultant next week and will raise the subject	OK	No data	Need some guidance to conduct exercise	Anxiety	Anxiety	Positive (-)	No data	Anxiety<br>Positive
Symptoms you have experienced over the past week	General back pain.<br>Tiredness	General back pain.<br>Tiredness	General back pain.<br>Tiredness	No data	Tiredness and pain	Pain<br>Fatigue	Pain<br>Fatigue	Pain<br>Fatigue	No data	Pain<br>Fatigue
Medications you have taken this week	Paracetamol	Paracetamol	Paracetamol	No data	As prescribed	Awareness	Awareness	Awareness	No data	Awareness
Healthcare appointments this week	None	None	Specialist 6 week follow up	No data	Limited access	No data	No data	Access	No data	Access
Rehabilitation progress this week<br>- *Your ability to exercise and do activity this week*<br>*Return to work / activities / hobbies as relevant to you*	Taking it easy with no lifting as directed	Taking it easy with no lifting as directed	Taking it easy with no lifting as directed<br>I am advised to take no activity beyond walking and no physiotherapy before next appointment in June!	No data	Advice and support given to exercise appropriately	Movement	Movement	Movement<br>Support	No data	Movement<br>Support
Positive thought(s) this week	Great relief to be making progress on sciatic pain at last	Great relief to be making progress on sciatic pain at last	Great relief to be making progress on sciatic pain at last	No data	Relief to be making progress	Positive (+)	Positive (+)	Positive (+)	No data	Positive

Participant: P15	Entry					Categories/Themes				
**Prompt**	**Week 1**	**Week 2**	**Week 3**	**Week 4**	**Summary**	**Week 1**	**Week 2**	**Week 3**	**Week 4**	**Combined themes**
Negative thought(s) this week	No data	No data	No data	No data	No data	No data	No data	No data	No data	No data
Most significant/memorable moment this week (may be positive or negative)	No data	No data	No data	No data	No data	No data	No data	No data	No data	No data
Please describe how this week has been compared to last week	Better	No data	No data	No data	Limited input	Positive (-)	No data	No data	No data	Positive
Please record any other comments you may like to make regarding how you have felt or your experiences over the past week?	No data	No data	No data	No data	No data	No data	No data	No data	No data	No data

Diary entry: P16

Participant: P16	Entry					Categories/Themes				
**Prompt**	**Week 1**	**Week 2**	**Week 3**	**Week 4**	**Summary**	**Week 1**	**Week 2**	**Week 3**	**Week 4**	**Combined themes**
Feelings about your progress this week	Steady progress over the left 4/5 weeks as expected	Still early days	Still a lot of leg pain	No data	Steady progress	Positive (+)	Speed	Pain	No data	Pain<br>Speed<br>Positive
Feelings about your recovery so far	Still feels slow, but to be expected	Slow but expected	My consultant says it could have up to 4 months<br>signs of recovery	No data	Advice and encouragement	Speed	Speed	Support	No data	Speed<br>Support
Level of motivation	Good positive mental approach	Still feeling positive	Weather not helping	No data	Overall positive	Positive (+)	Positive (+)	Mood	No data	Positive<br>Mood

Participant: P16	Entry					Categories/Themes				
**Prompt**	**Week 1**	**Week 2**	**Week 3**	**Week 4**	**Summary**	**Week 1**	**Week 2**	**Week 3**	**Week 4**	**Combined themes**
					outlook to recovery					
Symptoms you have experienced over the past week	Unable to do much warm weather	Back pain eased still leg pains	No data	No data	Restricted movement due to inclement whether	Movement	pain	No data	No data	Movement Pain
Medications you have taken this week	Painkiller – warfarin	Usual medication prescription enclosed	Usual	No data	As prescribed	Adherence	Adherence	No data	No data	Adherence
Healthcare appointments this week	Physio on 11.10.18. trying hand held crutches to keep me more upright	INR was 2.7-3mg by day. My consultant please with progress	None	No data	Access to healthcare	Access	Access	No data	No data	Access
Rehabilitation progress this week<br>- *Your ability to exercise and do activity this week*<br>*Return to work / activities / hobbies as relevant to you*	The weather has limited me for outside	Continuing with exercises	No data	No data	Continue with exercises but restricted by weather condition	Frustrated	Positive (+)	No data	No data	Frustrated Positive
Positive thought(s) this week	Still leg pain but different to back pain prior to operation	Positivity from My consultant	No data	No data	Staying positive because of advice from Surgeon	Pain	Positive (+)	No data	No data	Pain Positive
Negative thought(s) this week	No negative thoughts yet still early days	Seems slow	No data	No data	Slow but positive thoughts	Positive	Speed	No data	No data	Positive Speed
Most significant/memorable moment this week (may be positive or negative)	Physio supplies me with crutches to help with my posture	No data	Walked around meadow hall	No data	Support from healthcare professionals. Continue to walk	Positive (+)	No data	Movement	No data	Positive Movement
Please describe how this week has been compared to last week	Steady progress	No data	No data	No data	Steady progress	Positive (+)	No data	No data	No data	Positive

Participant: P16	Entry					Categories/Themes				
**Prompt**	**Week 1**	**Week 2**	**Week 3**	**Week 4**	**Summary**	**Week 1**	**Week 2**	**Week 3**	**Week 4**	**Combined themes**
Please record any other comments you may like to make regarding how you have felt or your experiences over the past week?	Early days	No data	No data	No data	Steady progress	Optimistic	No data	No data	No data	Optimistic

Diary entry: P17

Participant: P17	Entry					Categories/Themes				
**Prompt**	**Week 1**	**Week 2**	**Week 3**	**Week 4**	**Summary**	**Week 1**	**Week 2**	**Week 3**	**Week 4**	**Combined themes**
Feelings about your progress this week	I am feeling pain in my back	The pain is getting worse every day	The pain is still very bad and painful	The pain is still on my back and both legs	Experiencing pain	Pain	Pain	Pain	Movement Pain	Pain Movement
Feelings about your recovery so far	Very bad unable to walk properly and both feet are still swollen	None- still feeling pain on my bottoms, cannot sit down or lie down on the bed it is very painful	Still very weak	Feeling weak	Very slow recovery	Anxiety	Pain	Movement (-)	Movement (-)	Movement Pain Anxiety
Level of motivation	None	Very weak not much feeling, both legs are weak	None	None	Low motivation	Anxiety	Movement (-)	Anxiety	Anxiety	Anxiety Movement
Symptoms you have experienced over the past week	Not much without a stick	When going out I have to use the stick and my body is bending down look like my head and body is going down. My body	Walking outside with the help of a stick but today is going down forward	Still have to use a stick to walk- the body is bending feel like I am falling down	Restricted movement	Pain	Pain	Pain	Pain discomfort	Pain Discomfort

Participant: P17	Entry					Categories/Themes				
**Prompt**	**Week 1**	**Week 2**	**Week 3**	**Week 4**	**Summary**	**Week 1**	**Week 2**	**Week 3**	**Week 4**	**Combined themes**
		is bend down all the time								
Medications you have taken this week	Paracetamol 500mg, 2x3 a day, Senna 300mg 1 three times a day	Paracetamol 500mg 2x3 a day, Gabapentine 300mg 1x3 a day	Paracetamol, Gabapentine	Paracetamol, Gabapentine	As prescribed	Adherence Awareness	Adherence Awareness	Adherence Awareness	Adherence Awareness	Adherence Awareness
Healthcare appointments this week	Very painful and feeling weak and terrible in the morning (shaky)	Very bad experience and suffering	Very bad	Still the same pain	Limited as required	No data	No data	Access	Access	Access
Rehabilitation progress this week<br>- *Your ability to exercise and do activity this week*<br>*Return to work / activities / hobbies as relevant to you*	None	None	None	None	Walking at a reasonable pace	Anxiety	Positive (+)	Positive (+)	Positive (+)	Anxiety Positive
Positive thought(s) this week	None, doing exercise everyday don't work	None, doing exercise everyday don't work	None, doing exercise everyday don't work	None, doing exercise everyday don't work	Frustration, anxiety	Anxiety	Anxiety	Anxiety	Anxiety	Anxiety
Negative thought(s) this week	Negative	Negative	Negative	Negative	Negative	Anxiety	Anxiety	Anxiety	Anxiety	Anxiety
Most significant/memorable moment this week (may be positive or negative)	None	None	None	None	None	Anxiety	Anxiety	Anxiety	Anxiety	Anxiety
Please describe how this week has been compared to last week	Negative	Negative	Negative	Negative	Anxiety	Anxiety	Anxiety	Anxiety	Anxiety	Anxiety
Please record any other comments you may like to make	Getting worse	Still feeling weak and pain like some metal part	Still unable to go out alone but have to go out	Still unable to walk I got feeling I am	Overall related to pain	Mood	Pain	Pain	Support	Mood Pain Support

Participant: P17	Entry					Categories/Themes				
**Prompt**	**Week 1**	**Week 2**	**Week 3**	**Week 4**	**Summary**	**Week 1**	**Week 2**	**Week 3**	**Week 4**	**Combined themes**
regarding how you have felt or your experiences over the past week?		hurting me and I can't	with my wife and walk with a stick after a couple of sheet. My body bending down and I cannot walk straight as I used to walk	falling down on my face the last time I had physio was in Nov 2018	and discomfort					

Diary entry: P18

Participant: P18	Entry					Categories/Themes				
**Prompt**	**Week 1**	**Week 2**	**Week 3**	**Week 4**	**Summary**	**Week 1**	**Week 2**	**Week 3**	**Week 4**	**Combined themes**
Feelings about your progress this week	Very happy with progress	Happy with my progress	No entry	Still the same	Good progress	Positive (+)	Positive (+)	No entry	Positive (+)	Positive Movement
Feelings about your recovery so far	I still have good and bad times. I'm getting frustrated that I cannot do more	A little frustrated when trying to out sock and shoes as I can't bend but otherwise ok	No entry	New year slow down as expected	Recovery going well and at time slow	Anxiety	Frustrated	No entry	Movement	Anxiety Pain Movement
Level of motivation	Good hence I 'm enjoying walking even in the rain	Still walking but got bump on Thursday and didn't do anything	No entry	Difficult	Motivation appears to be good	Positive (+)	Positive (+)	No entry	Anxiety	Positive Anxiety
Symptoms you have experienced over the past week	Ache in my back this week and tightness around stomach incision site	Back ache sat down let leg pain	No entry	No entry	Pain in back and leg	Pain	Pain	No entry	No entry	Pain
Medications you have taken this week	Paracetamol, diazepam	Paracetamol, diazepam	No entry	Same	As prescribed	Adherence Awareness	Adherence Awareness	Adherence Awareness	Adherence Awareness	Adherence Awareness

Participant: P18	Entry					Categories/Themes				
**Prompt**	**Week 1**	**Week 2**	**Week 3**	**Week 4**	**Summary**	**Week 1**	**Week 2**	**Week 3**	**Week 4**	**Combined themes**
Healthcare appointments this week	None	None	No entry	Physio	Limited	No data	No data	No data	Access	Access
Rehabilitation progress this week<br>- *Your ability to exercise and do activity this week*<br>*Return to work / activities / hobbies as relevant to you*	Walk twice a day need to stand up a lot this week did some drawing	Walk everyday twice a day walking faster each day	No entry	New exercises from physio	Walking at a reasonable pace	Positive (+)	Positive (+)	No data	Positive (+)	Anxiety Positive
Positive thought(s) this week	Improving walking well	Walked to park and back nice to hear birds	No entry	Decided to book a cruise	Walking better	Movement	Positive (+)	No entry	Positive(+)	Positive (+)
Negative thought(s) this week	Painful to sit down hard to get to sleep	Bing bumped into the blame by old women and jamming my back	No entry	No entry	Disturbed sleep pattern caused by pain	Pain	Frustration	No data	No data	Frustration Sleep Irritation
Most significant/memorable moment this week (may be positive or negative)	Being able to move up and down stairs very well	Being able to cook properly now without a stool	No entry	Hoping the prospect of cruise will help	Able to engage in activities	Positive	Positive (+)	No data	Positive	Mood Positive
Please describe how this week has been compared to last week	Improvement again good in a positive way	Getting better when I feel good I walk more which makes me feel less bad days	No entry	Slow must get back into a routine	Managing pain throughout	Improvement	Positive (+)	No entry	Positive (+)	Positive Pain
Please record any other comments you may like to make regarding how you have felt or your experiences over the past week?	I’m struggling with socks and shoes. I have to sit up straight or feel pain in back. I’ve had one moment	Someone needs to design a sock put a device which works! Incision is healing well,	No entry	No entry	Struggling with routine activities	Frustrated	Frustrated	No entry	No entry	Frustrated

**Participant: P18**	**Entry**					**Categories/Themes**				
**Prompt**	**Week 1**	**Week 2**	**Week 3**	**Week 4**	**Summary**	**Week 1**	**Week 2**	**Week 3**	**Week 4**	**Combined themes**
	crying not sure why but felt much better after	using bio oil to help								

Diary entry: P19

**Participant: P19**	**Entry**					**Categories/Themes**				
**Prompt**	**Week 1**	**Week 2**	**Week 3**	**Week 4**	**Summary**	**Week 1**	**Week 2**	**Week 3**	**Week 4**	**Combined themes**
Feelings about your progress this week	Out of hospital 2 days	Progress slow and uncomfortable	Doing a bit more walking round garden	Positive – had a day out in Broadway	Progress slow and uncomfortable at times	Positive (+)	Anxiety	Movement	Positive (+)	Positive Movement Anxiety
Feelings about your recovery so far	Slow each day	Can do little bit more now	Good – wound healing well but uncomfortable lying in bed	Good – had the dressing changed and the wound is clean and healing. Dressing came off on Thursday	Recovery going well and at time slow	Anxiety	Positive (+)	Positive (+)	Positive (+)	Anxiety Pain Anxiety Movement
Level of motivation	good	Good	Good	Good – did some Tesco shopping (v. tiring!)	Motivation appears to be good	Positive (+)	Positive (+)	Positive (+)	Positive (+)	Positive
Symptoms you have experienced over the past week	Soreness lump in back	V sore stretch skin round op site	V sore back pain in legs	Soreness; pain in legs from thigh to ankle in bed; back feels like stiff cardboard that doesn't want to	Generalised pain and soreness in legs and surgical site	Pain	Pain	Pain	Pain discomfort	Pain Discomfort

Participant: P19	Entry					Categories/Themes				
**Prompt**	**Week 1**	**Week 2**	**Week 3**	**Week 4**	**Summary**	**Week 1**	**Week 2**	**Week 3**	**Week 4**	**Combined themes**
				bend when getting into bed!						
Medications you have taken this week	Lactose, dehydrocodeine, paracetamol, bisoprolol, apixabin, senna	paracetamol, senna, dehydrocodeine, bisoprolol, lactose	Senna, lactu;lose, paracetamol, dehydrocodeine bisoprolol,	Cocodamol; paracetamol, Apixaban; bisoprolol	As prescribed	Adherence Awareness	Adherence Awareness	Adherence Awareness	Adherence Awareness	Adherence Awareness
Healthcare appointments this week	None	None	Dressing changed by nurse on Tuesday	GP's nurse on Tuesday to have dressing replaced. Said it could come off after a couple of days.	Limited as required	No data	No data	Access	Access	Access
Rehabilitation progress this week<br>- *Your ability to exercise and do activity this week*<br>*Return to work / activities / hobbies as relevant to you*	v-little	Gentle walk and garden two/three times a day<br><br>none	Doing more walking	Improving – did some walking round Broadway on Monday; quite a bit round Tesco on Wednesday; attended an evening meeting of Arts Centre on Monday	Walking at a reasonable pace	Anxiety	Positive (+)	Positive (+)	Positive (+)	Anxiety Positive
Positive thought(s) this week	Good care in hospital	No data	Please it is healing well	Had some nice visitors and good weather						
Negative thought(s) this week	Catheter an drain a nuisance	Hate the surgeon word!!! (his not mine)	Pain in legs up-setting	Slight depression at level of discomfort at night –	Slight psychological issues related to pain and discomfort	Irritation	Frustration	Pain	Sleep Anxiety	Frustration Sleep Anxiety Irritation

**Participant: P19**	**Entry**					**Categories/Themes**				
**Prompt**	**Week 1**	**Week 2**	**Week 3**	**Week 4**	**Summary**	**Week 1**	**Week 2**	**Week 3**	**Week 4**	**Combined themes**
				interrupting sleep	detrimental to sleep pattern					
Most significant/memorable moment this week (may be positive or negative)	Coming home	Can journey to Bromsgrove to visit friends- went well more comfortable than expected	None	Taking off the dressing – for good	Socialising and dressing removed	Positive	Mood	No data	Positive	Mood<br>Positive
Please describe how this week has been compared to last week	n/a	Probably more painful as I have to cope with normal life	On the whole a slight improvement	Not a lot of difference – just a little less sore	Managing pain throughout	No data	Pain	Positive (+)	Positive (+)	Positive<br>Pain
Please record any other comments you may like to make regarding how you have felt or your experiences over the past week?	This was the period of the operation and home for 2 days	No data	Seem to have developed a pain in both legs when in bed which wakes me up and keeps me awake for a while. Goes from my hips to me ankles.	Had a nice chat with you!	Overall related to pain and discomfort	Mood	No data	Pain	Support	Mood<br>Pain<br>Support

Diary entry: P20

Participant: P20	Entry					Categories/Themes				
**Prompt**	**Week 1**	**Week 2**	**Week 3**	**Week 4**	**Summary**	**Week 1**	**Week 2**	**Week 3**	**Week 4**	**Combined themes**
Feelings about your progress this week	Better and quicker than expected. I had expected to be more restricted in movement	Initially this week feeling were good but as I have begun to have some leg pain this has resulted in apprehension and fear	A rollercoaster of a week and a real lack of energy initially, demotivated still frightened leg pain but relief when this began to subside	No data	Steady recovery and anxious of recurring pain	Movement	Anxiety<br>Pain	Energy	No data	Energy<br>Pain<br>Anxiety
Feelings about your recovery so far	Happy with progress so far. Although when pain sets it when waiting for meds in hospital it was a bit of a rollercoater	Started the week well, mobility increased very well but later in the week have started to have some leg pain	No data	No data	Steady progress over the week	Positive (+)	Pain<br>Anxiety	No data	No data	Positive<br>Anxiety
Level of motivation	I have been keen to get up and get moving	Still keen to try and get a balance between doing light work and walking and resting	Poor initially until Thursday- no energy but this changed overnight got up caught a bus and went into town	No data	Feeling energised and keen to engage in activities	Positive (+)<br>Movement	Fluctuate	Energy	No data	Positive<br>Energy<br>Fluctuate
Symptoms you have experienced over the past week	Pain, tiredness poor memory, irritability, emotional	Decrease in back pain better mobility increased leg pain	Leg pain initially but began to subside after couple of days – soreness is back	No data	Sustained pain over the next couple of weeks	Pain<br>Fatigue	Movement	Pain<br>Positive	No data	Pain<br>Positive<br>Movement
Medications you have taken this week	Paracetamol, dihydrocodeine, lactose, senna, oramorph, amitriptyline, omeprazole, vit D, Gabatenpin, naproxen	Paracetamol, dihydrocodeine, lactulose, oramorph, amitryptiline, omeprazole, gaberpentine, naproxen,	Paracetamol, dihydrocodeine, lactulose, oramorph, amitryptiline, omeprazole, gaberpentine, naproxen,	No data	As prescribed	Adherence	Adherence	Adherence	No data	Adherence

Participant: P20	Entry					Categories/Themes				
**Prompt**	**Week 1**	**Week 2**	**Week 3**	**Week 4**	**Summary**	**Week 1**	**Week 2**	**Week 3**	**Week 4**	**Combined themes**
Healthcare appointments this week	None	None	Nurse appointment at GP surgery to remove dressing and check wound – Monday.	No data	Limited health care access	No data	No data	Access	No data	Access
Rehabilitation progress this week<br>- *Your ability to exercise and do activity this week*<br>*Return to work / activities / hobbies as relevant to you*	Moving around better than expected- ability to turn over in bed by the end of this week.	Able to walk much further this week and feeling more agile generally day out in western-super-mare.	Real lack of energy initially but when this increased caught bus walked around supermarket	No data	Steady recovery, better movement	Movement	Movement Speed	Energy	No data	Energy Movement Speed
Positive thought(s) this week	I am happy with my progress – no leg pain	Initially in the week feeling positive, happy relieved	Relief that leg pain subsided	No data	Recovery going well	Positive (+)	Positive (+)	Mood	No data	Positive Mood
Negative thought(s) this week	Upset when I was waiting for medication in hospital and not feeling like I could stand the pain any longer	Later in the week feeling of concern and being frightened that leg pain is returning	Low mood and not being able to get motivated due to lack of energy	No data	Feeling upset and deflated because of the thought of leg pain returning	Anxiety	Anxiety Mood	Mood	No data	Anxiety Mood
Most significant/memorable moment this week (may be positive or negative)	Being home makes so much difference no leg pain!!!	Leg pain occurring	Leg pain went away	No data	Managing pain	Pain	Pain	Pain	No data	Pain
Please describe how this week has been compared to last week	N/A	Although I was less mobile and agile last week I feel more positive about last week as this week has been overshadowed by	A week of 2 halves – however because the leg pain has gone away I feel more positive and hopeful of a good recovery	No data	Pain impacting recovery	No data	Movement Pain	Positive (+)	No data	Movement Pain

Participant: P20	Entry					Categories/Themes				
**Prompt**	**Week 1**	**Week 2**	**Week 3**	**Week 4**	**Summary**	**Week 1**	**Week 2**	**Week 3**	**Week 4**	**Combined themes**
		returning of some leg pain								
Please record any other comments you may like to make regarding how you have felt or your experiences over the past week?	I have ended the week feeling positive however the week has been a rollercoaster of emotion pain and varying degrees of positivity	Returning leg pain has caused a dilemma… is this the norm for this operation? Will it go away? Is it going to get worse? Should I contact anyone for advice? Who should I contact? Will I look a fool if I do? I'm frightened and not sure what to do!	Can't wait to be able to drive and get out a bit more!	No data	Overall progressive recovery but frustrated with the lack of follow-up care	Positive (+)	Pain Frustration	Positive (+)	No data	Pain Positive Frustration

Diary entry: P21

Participant: P21	Entry					Categories/Themes				
**Prompt**	**Week 1**	**Week 2**	**Week 3**	**Week 4**	**Summary**	**Week 1**	**Week 2**	**Week 3**	**Week 4**	**Combined themes**
Feelings about your progress this week	Feeling good at moving around. Very worried about my wound leaking	Mobility not an issue	Wound now closed so v pleased	No data	Positive about recovery	Positive (+)	Movement	Positive (+)	No data	Positive Movement
Feelings about your recovery so far	I'm worried and unsure of wound healing	Still in hospital and now IV antibiotics	Still in limbo v. upset as no news not been referred to BIU!	No data	Frustration about recovery and referral	Anxiety	Frustration	Frustration	No data	Anxiety Frustration
Level of motivation	Good	Very low. Awaiting return to surgery tomorrow for wash out	Bad	No data	Wound affecting psychological well-being	Positive (+)	Frustration	Frustration	No data	Positive Frustration

Participant: P21	Entry					Categories/Themes				
**Prompt**	**Week 1**	**Week 2**	**Week 3**	**Week 4**	**Summary**	**Week 1**	**Week 2**	**Week 3**	**Week 4**	**Combined themes**
Symptoms you have experienced over the past week	Spine is v. painful wound leaking continually	Wound v sore with vacuum on it	pain	No data	Painful wound	Pain	Pain	Pain	No data	Pain
Medications you have taken this week	Oxynorm, dihydrocodeine, paracetamol, antibiotics	IV antibiotoics, oxynorm, dihydrocodeine, paracetamol	Oxynorm, dihydrocodeine paracetamol	No data	As prescribed	Adherence	Adherence	Adherence	No data	Adherence
Healthcare appointments this week	In hospital	Return to surgery 14/5 for wash out and sealing wound	In hospital	No data	In hospital stay	Access	Access	Access	No data	Access
Rehabilitation progress this week<br>- *Your ability to exercise and do activity this week*<br>*Return to work / activities / hobbies as relevant to you*	Wondering about room as isolated	No data	No data	No data	Anxious about infection and being isolated	Anxiety	No data	No data	No data	Anxiety
Positive thought(s) this week	Glad I'm in hospital so infection can be treated	Hope this is over soon	No data	No data	Relieved and positive	Mood	Positive (+)	No data	No data	Mood Positive
Negative thought(s) this week	Anxious and worried about how long I'll be in hospital (5-6 weeks)	How much longer	When this will end	No data	Worried about length of hospital stay	Anxiety	Anxiety	Depression	No data	Anxiety Mood
Most significant/memorable moment this week (may be positive or negative)	Returning to surgery for wash out and vacuum fitted	No data	No data	No data	Access to health care	Access	No data	No data	No data	Access
Please describe how this week has been compared to last week	Very bad lots of worry about the level of infection	No data	v. bad , v. emotional	No data	Emotionally draining, worried about	Anxiety	No data	Frustration	No data	Anxiety Frustration

Participant: P21	Entry					Categories/Themes				
Prompt	Week 1	Week 2	Week 3	Week 4	Summary	Week 1	Week 2	Week 3	Week 4	Combined themes
	and how it could affect me				wound getting infected					
Please record any other comments you may like to make regarding how you have felt or your experiences over the past week?	No data	No data	So upset with lack of communication no referral made to BIU so another week delay	No data	No clear care pathway	No data	No data	Frustration	No data	Frustration

Diary entry: P22

Participant: P22	Entry					Categories/Themes				
Prompt	Week 1	Week 2	Week 3	Week 4	Summary	Week 1	Week 2	Week 3	Week 4	Combined themes
Feelings about your progress this week	Early days yet, still experiencing pain from initial surgery.	Positive, making progress in moving around at home.	Positive, now moving around at home without crutches.	No data	Making positive progress and mobility improving	Pain	Movement	Movement	No data	Pain Movement
Feelings about your recovery so far	Short term pain, long term gain!!	Positive.	Very positive	No data	Feeling positive	Positive (+)	Positive (+)	Positive (+)	No data	Positive
Level of motivation	Good, with the exception of Sunday when suffering a migraine attack.	Good, was able to leave hospital on Monday 5th	Remains good on a day to day basis	No data	Reasonable level of motivation	Positive (+)	Positive (+)	Positive (+)	No data	Positive
Symptoms you have experienced over the past week	Pain at wound site. Left sided back pain (into buttock)	Pain at wound site. Left sided back pain (into buttock)	Pain at wound site. Left sided back pain (into buttock)	No data	Consistent pain around wound site	Pain	Pain	Pain	No data	Pain
Medications you have taken this week	Morphine (weaned off mid-week) Oxycodone Hydrochloride	Oxycodone Hydrochloride Tramadol Paracetamol	Oxycodone Hydrochloride Tramadol Paracetamol	No data	As prescribed and managing as appropriate	Adherence Awareness	Adherence Awareness	Adherence Awareness	No data	Adherence Awareness

Participant: P22	Entry					Categories/Themes				
**Prompt**	**Week 1**	**Week 2**	**Week 3**	**Week 4**	**Summary**	**Week 1**	**Week 2**	**Week 3**	**Week 4**	**Combined themes**
	Tramadol<br>Paracetamol<br>Meloxicam<br>Lansoprazole<br>Candesarten<br>Fluoxetine<br>Sumatriptan	Meloxicam<br>Lansoprazole<br>Candesarten<br>Fluoxetine<br>Sumatriptan	Meloxicam<br>Lansoprazole<br>Candesarten<br>Fluoxetine<br>Sumatriptan							
Healthcare appointments this week	Physiotherapist in hospital	Physiotherapist in hospital	Physiotherapist – xxx General Hospital<br>Nurse - xxx Clinic (wound check)	No data	Adequate access to health care	Access	Access	Access	No data	Access
Rehabilitation progress this week<br>- *Your ability to exercise and do activity this week*<br>*Return to work / activities / hobbies as relevant to you*	Mobilised from bed to bathroom on ward, using frame & toilet riser.<br>Able to shower independently in bathroom on ward.	Mobilised to be able to slowly walk length of the ward.<br>Discharged from hospital to home address, moving around with crutches within the home.	Able to walk around at home without the aid of crutches<br>2 trips out in car with partner – on both occasions, had meal out (unable to sit for entire duration of these, necessary to get up & move around)	No data	Reasonably mobile	Movement	Movement<br>Speed	Movement<br>Speed	No data	Movement<br>Speed
Positive thought(s) this week	I WILL be out of hospital by Wednesday 7th!	Made it home 2 days before I originally expected	Progress has been good, planning other short trips out. Looking forward to building up to unaccompanied walks for a short distance.	No data	Home comfort and planning trips	Positive (+)	Speed	Positive (+)	No data	Positive<br>Speed

Participant: P22	Entry					Categories/Themes				
**Prompt**	**Week 1**	**Week 2**	**Week 3**	**Week 4**	**Summary**	**Week 1**	**Week 2**	**Week 3**	**Week 4**	**Combined themes**
Negative thought(s) this week	Lack of sleep/ level of noise on ward. Nothing negative regarding procedure or progress/ rehab.	Lack of sleep/ disturbed due to pain when turning over in bed at night. Nothing negative regarding procedure or progress/ rehab.	Still lack of sleep/ disturbed due to wound pain when turning over in bed at night, although this has reduced from previous week.	No data	Disturbed sleep pattern	Sleep	Sleep	Sleep	No data	Sleep
Most significant/memorable moment this week (may be positive or negative)	Showering independently (Sat 3rd)	Walked downstairs without crutches.		No data	Independent	Movement	Movement	Movement Positive (+)	No data	Movement
Please describe how this week has been compared to last week	First week post surgery, no real comparison. Feeling very positive as no longer feeling leg pain post op which was significant problem pre-op.	Difference in mood being at home rather than in hospital – feeling much more positive being back in home environment.	Pain & stiffness in back has significantly reduced since last week, much easier to move around especially from sitting to standing. Use of crutches outside the home has primarily been to aid balance rather than needing to lean on for support as previously.	No data	Adequate recovery following surgery	Positive (+)	Mood	Pain	No data	Pain Mood Positive
Please record any other comments you may like to make regarding how you have felt or your	Mood took a significant dip on Sunday a.m. with suffering migraine attack & mobilizing very	I feel that it has been important now at home, to make sure that I keep some	Still maintaining structure to my day & ensured I have got up & dressed. Bath	No data	Adapting following surgery, activities of daily living affected as is	Mood	Positive (+)	Mood Energy	No data	Positive Energy Mood

Participant: P22	Entry					Categories/Themes				
**Prompt**	**Week 1**	**Week 2**	**Week 3**	**Week 4**	**Summary**	**Week 1**	**Week 2**	**Week 3**	**Week 4**	**Combined themes**
experiences over the past week?	little during the day.<br>However, physio attended Sunday also, since I was determined to get moving. Tried crutches & able to walk the length of the ward. Also stair assessment to evaluate mobility pre discharge.<br>Experienced additional pain & stiffness overnight, either due to lack of movement in a.m. or additional movement p.m.	structure to my day.<br>There have been varying times when I have woken/got out of bed, but have ensured that I freshened up & got dressed rather than sit in pyjamas all day. (Unable to shower as still waiting for bath board to be delivered by equipment services.<br>I also feel it's important to vary where I sit/ lie/ move - I have 3 main positions – upright in easy chair/ slightly reclined in easy chair/ horizontal on sofa & make sure I get up & move around regularly.	board arrived from equipment services which made quite a difference - such a mood boost to be able to get showered properly.<br>Main achievement of the week was being able to prepare a light meal with no assistance. It might have taken nearly 3 hrs. to prep & cook but the sense of satisfaction afterwards was brilliant, despite being very tired afterwards.		psychological wellbeing					

Non-consecutive week – (26/08/18 – 23/09/18) – 2 weeks difference between wk1 and wk2

Diary entry: P23

**Participant: P23**	**Entry**					**Categories/Themes**				
**Prompt**	**Week 1**	**Week 2**	**Week 3**	**Week 4**	**Summary**	**Week 1**	**Week 2**	**Week 3**	**Week 4**	**Combined themes**
Feelings about your progress this week	If progress is pain relief then its good	Slow	No progress and back to frustration	More positive after physio appointment	Steady progress	Pain Anxiety	Energy	Anxiety	Positive (+)	Anxiety Pain Energy Positive
Feelings about your recovery so far	Pain has eased and mobility has improved	positive	Delay in recovery	Better	Steady recovery	Movement Pain	Positive (+)	Anxiety	Positive (+)	Positive Pain
Level of motivation	high	High but tested	High but tested	High but tested	High level of motivation	Positive (+)	Positive (+)	Positive (+)	Positive (+)	Positive
Symptoms you have experienced over the past week	Continued lower back pain but greatly reduced leg pain	Bleeding from area of incision	Realistic pain levels due to personal decision to reduce medication with extra pain being introduced since new exercises have been introduced	Continued realistic pain levels due to personal decision to reduce medication but mainly in the area of the procedure	Managing pain by controlling and reducing pain killers	Pain	Anxiety	Pain	Pain	Pain Anxiety
Medications you have taken this week	Co-codamol,Ibuprofen,gabapentin and bisacodyl	Co-Codamol, Ibuprofen and Gabapentin	Co-codamol	Co-codamol	As prescribed	Adherence	Adherence	Adherence	Adherence	Adherence

Participant: P23	Entry					Categories/Themes				
**Prompt**	**Week 1**	**Week 2**	**Week 3**	**Week 4**	**Summary**	**Week 1**	**Week 2**	**Week 3**	**Week 4**	**Combined themes**
Healthcare appointments this week	GP for pain relief	A&E for attention to bleeding<br>G P for trimming of stitches	None	Physio	Adequate access to health care	Access	Access	No data	Access	Access
Rehabilitation progress this week<br>- *Your ability to exercise and do activity this week*<br>*Return to work / activities / hobbies as relevant to you*	Am able to carry out all advised exercise but a feeling i could be doing more	Exercise as advised in booklet from hospital	None	slight	Following advice and adhering to exercise regime	Speed	Movement	No data	Positive (+)	Positive<br>Speed<br>Movement
Positive thought(s) this week	Its getting better	The future	None	More hopeful	Hopeful for the future	Positive (+)	Positive (+)	No data	Positive (+)	Positive
Negative thought(s) this week	frustration	Frustration	Lack of support	Lack of support	General lack of support	Frustration	Frustration	Support (-)	Support (-)	Frustration<br>Support
Most significant/memorable moment this week (may be positive or negative)	Had my longest walk	Bleeding from incision left me feeling more frustrated over slow progress	Having to stop home exercise and walking because of extra pain it has induced	Having to stop home exercise because of extra pain it has induced	Setting goals and achieving milestones	Speed	Frustrated	Pain<br>Anxiety	Pain<br>Anxiety	Speed<br>Pain<br>Anxiety
Please describe how this week has been compared to last week	Much better	A little more mobility otherwise about the same	Total reverse as everything has gone backwards	Minimal change	Good progress and mobility improving	Positive (+)	Movement<br>Frustration	Speed	Positive (+)	Positive<br>Speed

**Participant: P23**	**Entry**					**Categories/Themes**				
**Prompt**	**Week 1**	**Week 2**	**Week 3**	**Week 4**	**Summary**	**Week 1**	**Week 2**	**Week 3**	**Week 4**	**Combined themes**
Please record any other comments you may like to make regarding how you have felt or your experiences over the past week?	Continued gradual progress mixed with frustration over how to progress when i have been told not to sit for long periods stand for long periods lift, bend, twist, pull, push or drive. What am I supposed to do for 16 hours a day to assist improvement (lack of post op advice)<br>No diary was recorded after hospital discharge last Friday evening, as lack of medication left me with severe pain and extreme constipation until after GP visit on Monday Please advise if more detailed responses are required on answers to diary prompts	No data	As in the past the lack of a specific person to contact means that with a physio/exercise problem I must wait 2 weeks for a resolution and as such this relates to 2 weeks lost recovery and an increase in personal frustration	As in the past the lack of a specific person of contact means that with a physio/exercise problem I must wait weeks for a resolve	Lack of structured care pathway	Anxiety<br>Frustration	No data	Frustration	Frustration	Anxiety<br>Frustration

Non-consecutive week – (12/09/18 – 30/12/18) – 2 weeks difference between wk1 and wk2

Diary entry: P24

Participant: P24	Entry					Categories/Themes				
**Prompt**	**Week 1**	**Week 2**	**Week 3**	**Week 4**	**Summary**	**Week 1**	**Week 2**	**Week 3**	**Week 4**	**Combined themes**
Feelings about your progress this week	Good progress. Have struggled more with the after effects of surgery and taking Medication than the surgery. Had the best result I could have had, the surgery has left me leg pain free and has corrected some posture/stature problems as a bonus.	Very positive, every day is a better day.	Good progress, some days I push more to walk further other days I rest quite a bit so hopefully I don't do too much.	Good progress again with everything getting easier.	Steady progress and hopeful recovery will be successful	Positive (+)	Positive (+)	Energy	Positive (+)	Positive Energy
Feelings about your recovery so far	Very, very good.	Excellent, no problems.	All positive.	Brilliant recovery, particularly considering it is 4 weeks post op.	Positive attitude to recovery	Positive (+)	Energy	Positive (+)	Positive (+)	Positive Energy
Level of motivation	High. Being leg pain free gives you a huge amount of motivation to now make the best of each day and hence your recovery.	Very high.	Still high due to being pain free.	Still really good.	Energy	Positive (+)	Positive (+)	Positive (+)	Positive (+)	Positive
Symptoms you have experienced over the past week	Sore wound, painful to lie on.	Aching in my back which very slight, not even	Wound is getting better, still gets a little	Slight back ache. Numbness in	Pain and discomfort associated	Pain	Pain	Pain Positive	Speed	Pain Positive Speed

Participant: P24	Entry					Categories/Themes				
**Prompt**	**Week 1**	**Week 2**	**Week 3**	**Week 4**	**Summary**	**Week 1**	**Week 2**	**Week 3**	**Week 4**	**Combined themes**
	Headaches - gradually getting less. Constipation. Minor pain around the bone areas that have had surgery.	requiring pain killers. Slight numbness remains in lower RH leg, nothing to complain about	swollen but responds well to icing. Have some aching in my back but I do feel it is to be expected.	lower leg. Feeling of back being uncomfortable which I can't say is pain, I just may be doing a little too much.	with surgery and medication					
Medications you have taken this week	Paracetamol Lactulose Senna	Nil	Occasional paracetamol and Naproxen.	Paracetamol Naproxen	As prescribed	Awareness	No data	Awareness	Awareness	Awareness
Healthcare appointments this week	None	Nurse to check wound and change dressing. All good.	None	None	Some health care intervention	Access	No data	No data	No data	Limited access
Rehabilitation progress this week<br>- *Your ability to exercise and do activity this week*<br>*Return to work / activities / hobbies as relevant to you*	Short walks - total around 3000 steps a day	Really good, have been increasing walking distance week each day with no effect on leg pain which is still zero. Longest walk about 2 miles. Total you this week for that day close to 4 miles. I love cooking and am now back in the kitchen and enjoying it. Couldn't stand and cook even a simple meal pre op. Going to the shops and being able to browse.	Walking further when I feel up to it. Keep having rest days.	Walking further, one day it was 5 miles over the course of the day. I get tired but think that's because it's a long time since I could walk anything like that.	Willingness to improve and help recovery	Speed	Speed	Speed	Speed Fatigue	Speed Fatigue

**Participant: P24**	**Entry**					**Categories/Themes**				
**Prompt**	**Week 1**	**Week 2**	**Week 3**	**Week 4**	**Summary**	**Week 1**	**Week 2**	**Week 3**	**Week 4**	**Combined themes**
Positive thought(s) this week	I am blessed to have such a good result and living without leg pain is the best thing ever. Other pain is incidental and easy to live with compared.	Life is great without pain! :-)	Life without leg pain is great!	Everything is going swimmingly well!	Managing pain and generally feeling good	Mood	Mood	Energy	Energy	Mood Energy
Negative thought(s) this week	Nothing really negative, you can't get over having no leg pain. Constipation was the worst thing.	None	Numbness in leg has increased a little which has disappointed me a little. Not enough to get upset about it though!	None.	Staying positive	Positive (+)	No data	Pain Positive (+)	No data	Positive Pain
Most significant/memorable moment this week (may be positive or negative)	Realising that the leg pain has gone - Mission Accomplished	Walking without pain, being able to stand up straight. Getting back in the kitchen!	Have reached nearly 4 miles walking a day pain free. Maximum before was 1.75 miles all in increasing pain.	Walking with friends and family with a smile on my face. It's been over ten years since I could do that!	Setting milestones and satisfaction in achieving them	Positive (+) Mood	Movement	Speed	Energy	Energy Speed Movement
Please describe how this week has been compared to last week	This week is heaven, last week was hell. Last week everything was a struggle, this week anything is achievable, within the constraints of recovering from surgery!	Last week was good, this week even better	A better week, wound is getting easier so must be getting better.	Even better, doing what I like when I want to without strategic planning makes you feel good about life.	Remarkable progress	Positive (++)	Energy	Positive (+)	Mood	Energy Mood Positive
Please record any other comments you	Being is hospital is one of the	Everything is a pleasure again -	Enjoying pushing myself	All positive looking	Pleasant recovery	Positive (+)	Positive (+)	Positive (+)	Positive (+)	Positive

Participant: P24	Entry					Categories/Themes				
**Prompt**	**Week 1**	**Week 2**	**Week 3**	**Week 4**	**Summary**	**Week 1**	**Week 2**	**Week 3**	**Week 4**	**Combined themes**
may like to make regarding how you have felt or your experiences over the past week?	worst experience I have ever had, I feel for the poor people in there who are poorly. Once discharged, I have put that behind me to concentrate on my recovery. It shouldn't be that way.	even grocery shopping! Really surprised at how little pain I have had in my back, I'd been warned I would experience back pain	a bit further each, just trying not to go too far.	forward to doing so much more than I have been able to						

Non-consecutive week – (16/09/18 – 07/10/18) – 2 weeks difference between wk1 and wk2

Diary entry: P25

Participant: P25	Entry					Categories/Themes				
**Prompt**	**Week 1**	**Week 2**	**Week 3**	**Week 4**	**Summary**	**Week 1**	**Week 2**	**Week 3**	**Week 4**	**Combined themes**
Feelings about your progress this week	Frustrated	It has been a difficult week in regards to progress made. I am still suffering with nerve pain in the foot and leg. this has resulted in a trip to the doctor	It has been a difficult week with the nerve pain overshadowing every thing	No data	Frustrated because of constant pain	Frustrated	Pain	Pain	No data	Pain Frustration
Feelings about your recovery so far	good	As above with regards to the nerve pain. The pain round the back and wound has eased	Still hopeful of a full recovery, there has been slight improvement in my symptoms. feeling frustrated as was hoping to be further	No data	Hopeful of a full recovery	Positive (+)	Pain	Frustration	No data	Positive Pain

Participant: P25	Entry					Categories/Themes				
**Prompt**	**Week 1**	**Week 2**	**Week 3**	**Week 4**	**Summary**	**Week 1**	**Week 2**	**Week 3**	**Week 4**	**Combined themes**
			down the recovery road							
Level of motivation	High	Low, tired due to not sleeping during the week.	. Feeling pretty flat. As frustration seems to be driving everything.	No data	Frustrated by lack of progress	Mood	Positive (-)	Frustration	No data	Positive Frustration
Symptoms you have experienced over the past week	Back and knee pain	Tender feeling in my foot with increased nerve pain in the foot and leg. It has resulted in my mobility being restricted. At times it has resulted in me not being able to tolerate a shoe on the foot.	Back pain seems to be under control with symptoms being stiff back. Nerve pain is still the main issue for me. This goes from the right outer leg into the foot	No data	Recurrent pain and activities of daily living restricted	Pain	Pain Frustration	Positive (+)	No data	Pain Positive Frustration
Medications you have taken this week	Paracetamol and ibrufen. for knee pain	Amitriptyline 25mg*2 tablets daily Amlopodine 10mg daily Atorstatin 20mg daily Bendroflumethiaide 2.5mgdaily Doxazosin 2mg daily Folic acid 5mg daily except day of injection Hydroxychloroquine 200mg * 2 daiy Naproxen 500mg * 2 daily Pantaprazole 20mg daily Pitzotifen 1.5mg daily Pregablin 75mg *4 twice daily (increased this week) Ramipril 10mg daily Sulfasalazine 500mg * 3 twice daily Tramadol 50mg *2 three times a day (increased this	Amitriptyline 25mg*2 tablets daily Amlopodine 10mg daily Atorstatin 20mg daily Bendroflumethiaide 2.5mgdaily Doxazosin 2mg daily Folic acid 5mg daily except day of injection Hydroxychloroquine 200mg * 2 daiy Naproxen 500mg * 2 daily Pantaprazole 20mg daily Pitzotifen 1.5mg daily Pregablin 75mg *4 twice daily (increased this week) Ramipril 10mg daily	No data	As prescribed and when needed according to pain threshold	Adherence Awareness	Adherence Awareness	Adherence Awareness	No data	Adherence Awareness

Participant: P25	Entry					Categories/Themes				
**Prompt**	**Week 1**	**Week 2**	**Week 3**	**Week 4**	**Summary**	**Week 1**	**Week 2**	**Week 3**	**Week 4**	**Combined themes**
		week) Mirtazapine 15mg daily Methotrexate 20mg inject once per week Oramorph	Sulfasalazine 500mg * 3 twice daily Tramadol 50mg *2 three times a day (increased this week) Mirtazapine 15mg daily Methotrexate 20mg inject once per week Oramorph							
Healthcare appointments this week	None	Appointment at doctors due to nerve pain in foot. Increased tramadol and oramorph. Doctor is ringing in two weeks to check progress	I was due to see the consultant, however two letters came with different dates on, I picked the last one received and it was the wrong one. Took me two hours to get there and the same back. Another appointment has been received	No data	Fragmented follow-up care	No data	Access	Restricted	No data	Access Restricted
Rehabilitation progress this week<br>- *Your ability to exercise and do activity this week*<br>*Return to work / activities / hobbies as relevant to you*	It has been our sale and change over from summer to winter wear which has been a challenge both physically and mentally	Still absent from work. I have tried to mobilise by walking as directed by physio. Last appointment they advised walking only	Sticking with walking as advised by physio. They advised me to keep my back stiff to allow the plates to set and fuse	No data	Steady progress	Mood	Anxiety	Positive (+)	No data	Mood Anxiety Positive
Positive thought(s) this week	Did it	That there is a little more feeling in my right foot on three of my toes	Booked a week away, so depart on 14th October	No data	Steady progress and improved social life	Positive (+)	Movement	Social	No data	Positive Movement Social
Negative thought(s) this week	But it was too much for me	Although there is feeling in part of the foot, the rest of the foot	The feeling of being a failure and a burden, as still not driving. Suffer from	No data	Surgery and recovery period has had a	Energy	Pain	Emotion	No data	Energy Pain Emotion

Participant: P25	Entry					Categories/Themes				
**Prompt**	**Week 1**	**Week 2**	**Week 3**	**Week 4**	**Summary**	**Week 1**	**Week 2**	**Week 3**	**Week 4**	**Combined themes**
		is feeling more tender and sore.	depression and have been in a black hole. It doesn't help as my work friend is away on holiday. Since being off usually phone or call for a coffee with him. The holiday is a lift, however usually book a villa or go to my sister in laws villa, but with me not driving wasn't feasible. Managed to find some nice apartments in a great location. Got to rely on people to take me to the airport		negative impact on psychological well-being					
Most significant/memorable moment this week (may be positive or negative)	Doing the change over	None	Improvement in the nerve pain in my foot. The area affected is getting smaller. Think that the oromorph is helping to control this. I have been using morphine just at night. Also been sleeping for longer periods, however still up a big part of the night	No data	Adjusting following surgery and managing pain	Mood	No data	Sleep<br>Pain	No data	Sleep<br>Pain
Please describe how this week has been compared to last week	A lot of challenges	Difficult to say as there is more feeling in part of the foot, but the rest of the foot is a lot more	Being able to do things without having to think about whether me	No data	Recovery appears to be a challenge	Depression<br>Frustration	Pain	Pain	No data	Pain<br>Emotion

Participant: P25	Entry					Categories/Themes				
**Prompt**	**Week 1**	**Week 2**	**Week 3**	**Week 4**	**Summary**	**Week 1**	**Week 2**	**Week 3**	**Week 4**	**Combined themes**
		tender. The back area and around the wound is healing nicely	leg's going to be right, or have I got it, have I just twisted it the wrong way or – you know							
Please record any other comments you may like to make regarding how you have felt or your experiences over the past week?	None	None	None	No data	No data	No data	No data	No data	No data	No data

Non-consecutive week – (14/10/18 – 04/11/18) – 2 weeks difference between wk1 and wk2

Diary entry: P26

**Participant: P26**	**Entry**					**Categories/Themes**				
**Prompt**	**Week 1**	**Week 2**	**Week 3**	**Week 4**	**Summary**	**Week 1**	**Week 2**	**Week 3**	**Week 4**	**Combined themes**
Feelings about your progress this week	Definitely frustrated. I know this is going to take a long time to recover from, but I'm an active guy and not being able to do the most basic things like do the washing up, or shower without excruciating pain is infuriating. Weirdly it doesn't make me mad or angry	On the whole, I don't really feel like I've changed much since last week. I went to go watch my home town football team on Saturday and that was tough. I wasn't in agony or anything like that, but the driving there, the walking to the stadium and the sitting in the cold for two hours just meant that I was exhausted	No data	No data	Trying to lead a normal life but impeded by constant pain	Pain	Pain<br>Fatigue	No data	No data	Pain<br>Fatigue
Feelings about your recovery so far	My body feels totally different. I worry that what's been done to correct my problem has caused issues with my legs because of the constantly fluctuating pain. I can handle having to walk like and old man for a while, but shaking with pain while I go to the loo is a new experience for me. I hope this gets better quickly. I do drink a lot of tea.	It is what it is. The drugs regime my doctor has me on helps numb the constant pain. I'm mobile enough to go get a pint of milk from the local shop, but I get tired walking up the slight hill. Little things like that mean I'm a bit down about how long this is going to take to be relatively normal,	No data	No data	Anxious about procedure and the unintended damage this may have caused. The pain and expected length of time until recovery is stressful	Anxiety<br>Pain	Stress<br>Anxiety<br>Mood	No data	No data	Stress<br>Anxiety<br>Pain<br>Mood

Participant: P26	Entry					Categories/Themes				
**Prompt**	**Week 1**	**Week 2**	**Week 3**	**Week 4**	**Summary**	**Week 1**	**Week 2**	**Week 3**	**Week 4**	**Combined themes**
		never mind going back to work.								
Level of motivation	I'm obviously up and down. Better since I ran out of drugs the hospital gave me though. I didn't like how they made me mentally. I'm a fairly positive and determined person in general but like I mentioned before, I'm worried. Will I ever be able to do the things I love again...who knows? My family have been very supportive, and I've suddenly gotten much better at Call of Duty. Every cloud.	It hasn't been as crazy up and down since I changed medication. I've had a lot of visitors this week so it's been largely positive.	No data	No data	Family/friend support, however level of anxiety has not subsided and psychological wellbeing is largely affected by uncertainty about recovery	Anxiety<br>Stress	Support	No data	No data	Anxiety<br>Support<br>Stress
Symptoms you have experienced over the past week	I struggle to stand still. I've got a fairly high tolerance for pain and I only managed 13 seconds when I challenged myself in the middle of last week. It takes my breath away. Showering and going to the loo entails some sort of dance. I can imagine it would be quite funny for people to watch. Walking doesn't cause me pain, but my mobility is clearly	I've had a runny nose, a sore throat and general lethargy. That's the cold I got from my little nephew. The sneezing part of it is painful. Suddenly jerking around uncontrollably is not good for my core. In general, the pain is managed by the drugs and it changes from	No data	No data	Generally feeling low in energy and disturbed sleep pattern. Aware of the pain that might be cause by sudden movement	Sleep<br>Anxiety<br>Fatigue<br>Mood	Fatigue<br>Pain	No data	No data	Pain<br>Fatigue<br>Anxiety<br>Sleep<br>Mood

Participant: P26	Entry					Categories/Themes				
Prompt	Week 1	Week 2	Week 3	Week 4	Summary	Week 1	Week 2	Week 3	Week 4	Combined themes
	limited. It'll take me a while to get used to my new configuration. Sleeping is a nightmare. It always was but now I can't take enough pain relief to get me through the night and I don't have a handy morphine button like post-op. So if I get 3-4 hours in before I'm woken up with fire down my legs then I'm doing well. Which means I'm exhausted throughout the day and with it costing me so much more energy to do anything, I'm simply knackered.	being a nerve pain to being a hip pain.								
Medications you have taken this week	Tramadol, Gabapentin, Solpadeine, Co-codamol, Codeine, Paracetamol I'm down to just the Solpadeine now. The others I found to be less effective. I mean what's the point of Gabapentin?	Solpadeine, Co-codamol, Gabapentin. That's it. Three times a day.	No data	No data	Dependent of the level of pain	Awareness	Awareness Adherence	No data	No data	Awareness Adherence
Healthcare appointments this week	I've had to re-register at the practice I had in Scotland upon my return home. So I won't get to see a	I've had a repeat prescription made for me over the phone.	No data	No data	Access to healthcare services is available but this needs to	Anxiety Access	Access	No data	No data	Anxiety Access

Participant: P26	Entry					Categories/Themes				
**Prompt**	**Week 1**	**Week 2**	**Week 3**	**Week 4**	**Summary**	**Week 1**	**Week 2**	**Week 3**	**Week 4**	**Combined themes**
	doctor until next week at the earliest. That'll be fun explaining all of this to the GP				be arranged by patients no clear treatment pathway					
Rehabilitation progress this week<br>- *Your ability to exercise and do activity this week*<br>*Return to work / activities / hobbies as relevant to you*	I can walk well enough. Uphill puts me down into first gear, but to the casual observer I think I hide my lack of mobility well. I've been stretching my leg muscles, and trying to relieve nerve tension down my thighs. Playing with my young niece and nephews has been fun, testing and tiring. I've managed to do some basic research and presenting of findings for work. But again, sitting for a long spell with a laptop is a tough ask. I'm a million miles away from being able to do anything physical related to work	Like I said, I've pushed myself with the walking longer distances, and driving. It frustrates me that I get tired so quickly. I work in outdoors/adventure sports, so there is no way I'd be able to return to work or do any of the things I enjoy. Currently my life is:- wake up, eat, watch TV, play playstation, maybe go for a walk, eat, play PlayStation, eat.... etc. etc. I'm sure that'll change in the next month or so. Oh I forgot to mention drinking tea. I drink a lot of tea.	No data	No data	Stress<br>Anxiety<br>Mood	Stress<br>Anxiety<br>Fatigue		No data	No data	Stress<br>Anxiety<br>Fatigue
Positive thought(s) this week	I'm walking, and I'm not on so many drugs. That has improved my mood dramatically. I was able to take the kids to the park yesterday. I sat on a bench for	I've been fit enough to host friends and play with my niece and nephews. I'm tired. To the bone tired. But it was worth it for all the positive	No data	No data	Some positive and improved psychological wellbeing	Positive (+)	Support<br>Mood	No data	No data	Support<br>Mood<br>Positive

Participant: P26	Entry					Categories/Themes				
Prompt	Week 1	Week 2	Week 3	Week 4	Summary	Week 1	Week 2	Week 3	Week 4	Combined themes
	most of it, but I got myself there and back. Small victories.	vibes and fun. Plus I got to see my mighty Raith Rovers play for the first time in well over a year.								
Negative thought(s) this week	When I woke up on Saturday I just wanted to scream and let all the frustration out. I think I was trying to tidy up my bedroom, but in the end I needed to call a friend and she stopped me from spiralling down into tears.	Just that the pain isn't subsiding, and the strength in my core is so pathetic I can't put socks on without some weird Pilates manoeuvre.	No data	No data	Pain and recovery is having a detrimental effect on psychological wellbeing	Mood<br>Frustration	Pain<br>Energy	No data	No data	Mood<br>Pain<br>Energy
Most significant/memorable moment this week (may be positive or negative)	Trying to stand while making tea. 13 seconds. I was shaking in pain and dizzy from it. But I made that cuppa, and I rewarded myself with a Hob-Nob!	I was able to play with my 1 year old nephew for a long time without me being sore or , more importantly, him not crying. That was the breakthrough moment for him and I. Living and working away from them is one of the hardest parts of my lifestyle.	No data	No data	Adjusting to life following surgery	Pain<br>Anxiety	Positive (+)	No data	No data	Positive<br>Anxiety<br>Pain
Please describe how this week has been compared to last week	Well last week I was mostly off my head on drugs, so ... clearer is the word I would use.	Much the same to be honest. Infinitely better than the week before due to the drugs mostly. They do make the	No data	No data	Better management of pain although the drugs are causing side effects such	Mood<br>Pain	Pain<br>Fatigue<br>Sleep	No data	No data	Mood<br>Pain<br>Fatigue<br>Sleep

Participant: P26	Entry					Categories/Themes				
**Prompt**	**Week 1**	**Week 2**	**Week 3**	**Week 4**	**Summary**	**Week 1**	**Week 2**	**Week 3**	**Week 4**	**Combined themes**
		pain manageable, but they also make me quite drowsy. I feel like I could fall asleep at any point in the day.			as fatigue, drowsiness					
Please record any other comments you may like to make regarding how you have felt or your experiences over the past week?	You guys doing this survey are the only folks who have checked up on me since I was unceremoniously booted out of the hospital nearly 2 weeks ago.	Overall, this week was a good week. Not because I got any better, but other factors brought happiness and fun to my life. I do wonder every day how long it's going to be before I get to go sailing again, and whether I'll ever be able to snowboard. It's going to be a long old slog of a recovery.	No data	No data	Non-existent treatment pathway	Access Frustration	Positive Anxiety	No data	No data	Frustration Access Anxiety

Non-consecutive week – (27/01/19 – 24/02/19) – 1-week difference between the two entries

Diary entry: P27

**Participant: P27**	**Entry**					**Categories/Themes**				
**Prompt**	**Week 1**	**Week 2**	**Week 3**	**Week 4**	**Summary**	**Week 1**	**Week 2**	**Week 3**	**Week 4**	**Combined themes**
Feelings about your progress this week	A little. Still taking pain killers for post op pain	Not a lot. Still ache quite a bit and still doing exercises daily	No data	No data	Pain and trying to cope	Pain	Pain	No data	No data	Pain
Feelings about your recovery so far	Overall very good. I have post op pain but the original symptoms have been dealt with	Good. The operation worked on the original pain but still hurt every day	No data	No data	Feeling better and pain is gradually subsiding	Positive (+)	Pain	No data	No data	Positive Pain
Level of motivation	7/10	High	No data	No data	High level of motivation	Energy	Energy	No data	No data	Energy
Symptoms you have experienced over the past week	Lower back pain when the pain relief wears off and if i walk or stand for too long. Certain very flat shoes make it hurt more. Still wake up during the night in pain when pain relief wears off	Lower rear back pain. Particularly by end of day	No data	No data	Managing pain but at times, particularly at night it's distressing	Pain	Pain	No data	No data	Pain
Medications you have taken this week	Cocodamol 30/500 × 1.5 every 6 hours	Cocodamol 30/500 ×1 4 times a day	No data	No data	As prescribed and dependent on pain threshold	Adherence	Adherence	No data	No data	Adherence

**Participant: P27**	**Entry**					**Categories/Themes**				
**Prompt**	**Week 1**	**Week 2**	**Week 3**	**Week 4**	**Summary**	**Week 1**	**Week 2**	**Week 3**	**Week 4**	**Combined themes**
Healthcare appointments this week	None	Physio for shoulder and review of back pain. Have said I'll give it a couple more months	No data	No data	Access to healthcare	No data	Access	No data	No data	Access
Rehabilitation progress this week<br>- *Your ability to exercise and do activity this week*<br>*Return to work / activities / hobbies as relevant to you*	I continue to do a form of pilates for 20- 25 mins Daily and stretches before work. They are getting easier but I have to be mindful not to overdo them	Back still clicks with exercises. Walking more but still pain	No data	No data	Engaged into structured exercises but conscious of limitation	Mobility	Pain	No data	No data	Mobility<br>Pain
Positive thought(s) this week	Thinking about a summer holiday and have bought some sturdy walking boots to take	Happier life/work balance. Nice new relationship. Booked a week's holiday in June	No data	No data	Support from inner circle, feeling psychologically better	Mood	Positive (+)	No data	No data	Mood<br>Positive
Negative thought(s) this week	How long will it take before I am pain free and don't have to do daily stretches. Would I undo any of the surgery if I slipped on ice?	Why is it still clicking- feels like a ratchet in my lower back. Worried plates/screws are loose.<br>Want to be pain free	No data	No data	Still struggling with pain and may some detrimental effect on psychological wellbeing	Anxiety	Anxiety	No data	No data	Anxiety
Most significant/memorable moment this week (may be positive or negative)	My daughter's wedding	Walked 5 KMs	No data	No data	Setting goals and awaiting happy events	Energy	Energy	No data	No data	Energy

Participant: P27	Entry					Categories/Themes				
**Prompt**	**Week 1**	**Week 2**	**Week 3**	**Week 4**	**Summary**	**Week 1**	**Week 2**	**Week 3**	**Week 4**	**Combined themes**
Please describe how this week has been compared to last week	About the same	Same	No data	No data	Appears to be getting back to normal	Positive (+)	Positive (+)	No data	No data	Positive
Please record any other comments you may like to make regarding how you have felt or your experiences over the past week?	My job is a driving examiner and my employer has kindly bought me a back support to take on tests. Unfortunately i have wrenched my shoulder to avoid an accident on test so finding some exercises difficult – i.e. No weight bearing	No data	No data	No data	Support from workplace is appreciated	Support	No data	No data	No data	Support

Diary entry: P28

Participant: P28	Entry					Categories/Themes				
**Prompt**	**Week 1**	**Week 2**	**Week 3**	**Week 4**	**Summary**	**Week 1**	**Week 2**	**Week 3**	**Week 4**	**Combined themes**
Feelings about your progress this week	Progress continues slowly	Progress continues slowly following last weeks great gain	Slow progress, better than last week	Feeling better less pain	Good progress but feels need more information	Slow	Progress	Progress	Feel better	Slow progress
Feelings about your recovery so far	Pleased with progress	Happy but could do with more guidance on exercise.	Please with progress, doing some exercise	Continue to do some exercise, but cautious	Feeling better and pain is gradually subsiding	Positive (+)	Pain	Progress	Progress	Positive
Level of motivation	OK	OK. Wish I knew what exercise to take. Seeing Consultant next week and will raise the subject	Feel better but need some more info	Better after I have spoken with my consultant	High level of motivation	Energy	Energy	Energy	Energy	Energy
Symptoms you have experienced over the past week	General back pain.<br><br>Tiredness	General back pain.<br><br>Tiredness	Persistent pain	Pian easing	Managing pain but at times, quite awful	Pain	Pain	Pain	Pian	Pain
Medications you have taken this week	Paracetamol	Paracetamol	Paracetamol	Paracetamol	As prescribed and dependent on pain threshold	Adherence	Adherence	Adherence	Adherence	Adherence

Participant: P28	Entry					Categories/Themes				
**Prompt**	**Week 1**	**Week 2**	**Week 3**	**Week 4**	**Summary**	**Week 1**	**Week 2**	**Week 3**	**Week 4**	**Combined themes**
Healthcare appointments this week	Specialist 6 week follow up	None	None	None	Access to healthcare	Follow-up	No access	No access	No access	Access
Rehabilitation progress this week<br>- *Your ability to exercise and do activity this week*<br>*Return to work / activities / hobbies as relevant to you*	Taking it easy with no lifting as directed<br><br>I am advised to take no activity beyond walking and no physiotherapy before next appointment in June!	Taking it easy with no lifting as directed	Active but cautious	Managing better	Engaged into structured exercises but conscious of limitation	Mobility	Pain	Active	Active	Mobility<br>Pain
Positive thought(s) this week	Great relief to be making progress on sciatic pain at last	Great relief to be making progress on sciatic pain at last	Slow progress	Happy with progress	Support from inner circle, feeling psychologically better	Mood	Positive (+)	Positive (+)	No data	Mood<br>Positive
Negative thought(s) this week	Slow progress	When will I return to some normality	Getting there but slow	Will I fully recover	Still struggling with pain and may some detrimental effect on psychological wellbeing	Anxiety	Anxiety	Distress	Distress	Anxiety<br>Distress
Most significant/memorable moment this week (may be positive or negative)	Less pain I suppose	Engaging better with family	Made a cuppa and pot around	Tried flight of stairs	Setting goals and awaiting happy events	Energy	Energy	Positive (+)	Energy	Energy

Participant: P28	Entry					Categories/Themes				
**Prompt**	**Week 1**	**Week 2**	**Week 3**	**Week 4**	**Summary**	**Week 1**	**Week 2**	**Week 3**	**Week 4**	**Combined themes**
Please describe how this week has been compared to last week	Better	Better than last	Getting there I believe	Lot better	Appears to be getting back to normal	Positive (+)	Positive (+)	Positive (+)	Positive (+)	Positive
Please record any other comments you may like to make regarding how you have felt or your experiences over the past week?	A lot of pain but felt surgery went okay	Slow progress and pain not easing	Better and pain is more manageable	Feels better and happy I suppose with myself	Progress was slow but determined to get better	Pain	Positive (+)	Positive (+)	Positive (+)	Positive
