# Supplementary material for: Patient journey following lumbar spinal fusion surgery (FuJourn): A multicentre exploration of the immediate post-operative period using qualitative patient diaries
Source: PLoS One. 2020 Dec 1;15(12):e0241931. doi: 10.1371/journal.pone.0241931 (PMC7707593; doi:10.1371/journal.pone.0241931)
Supplement: Attachment — Submitted filename: Response to reviewers PlosOne Diaries.docx [file pone.0241931.s003.docx]

5^th^ October 2020

Dear Panagiotis Kerezoudis

**Re:	PONE-D-20-20781**

**Patient journey following lumbar spinal fusion surgery (FuJourn):  a multicentre exploration of the immediate post-operative period using qualitative patient diaries**

Thank you for inviting us to submit a revised version of our manuscript addressing the points raised within the review process. Please find attached alongside this letter a copy of the revised manuscript with track changes and the clean manuscript as requested, along with a new Figure.

Please see tabulated below an outline of each change made-as raised in the reviewer comments, or our reasoning as to why it has not been possible to address a comment.

| **Points raised** | **Response from authors**  (Page no refers to annotated manuscript) |
| --- | --- |
| **Journal requirements** | |
| 1. Please ensure that your manuscript meets PLOS ONE's style requirements, including those for file naming. The PLOS ONE style templates can be found at https://journals.plos.org/plosone/s/file?id=wjVg/PLOSOne_formatting_sample_main_body.pdf and https://journals.plos.org/plosone/s/file?id=ba62/PLOSOne_formatting_sample_title_authors_affiliations.pdf | Thank you.  The manuscript has been reviewed to ensure all requirements are met. |
| 2. We note that you have indicated that data from this study are available upon request. PLOS only allows data to be available upon request if there are legal or ethical restrictions on sharing data publicly. For information on unacceptable data access restrictions, please see http://journals.plos.org/plosone/s/data-availability#loc-unacceptable-data-access-restrictions.  In your revised cover letter, please address the following prompts:  a) If there are ethical or legal restrictions on sharing a de-identified data set, please explain them in detail (e.g., data contain potentially identifying or sensitive patient information) and who has imposed them (e.g., an ethics committee). Please also provide contact information for a data access committee, ethics committee, or other institutional body to which data requests may be sent.  b) If there are no restrictions, please upload the minimal anonymized data set necessary to replicate your study findings as either Supporting Information files or to a stable, public repository and provide us with the relevant URLs, DOIs, or accession numbers. Please see http://www.bmj.com/content/340/bmj.c181.long for guidelines on how to de-identify and prepare clinical data for publication. For a list of acceptable repositories, please see <http://journals.plos.org/plosone/s/data-availability#loc-recommended-repositories>.  We will update your Data Availability statement on your behalf to reflect the information you provide. | All data has been provided and the original statement has been removed. |
| 3. Please include captions for your Supporting Information files at the end of your manuscript, and update any in-text citations to match accordingly. Please see our Supporting Information guidelines for more information: http://journals.plos.org/plosone/s/supporting-information | Thank you, captions are now included. |
| **Reviewer #1** | |
| The authors present a unique study of 28 patients who contributed weekly diaries in the first four weeks following short construct lumbar fusion. They identified three distinct trajectories of "meaningful recovery, progressive recovery, and disruptive recovery." This is an interesting study that may help with patient education of what to expect in the immediate postop period. Of course, no conclusions can be made about the efficacy of lumbar spine fusion surgery from this study as full recovery takes at least 3-6 months and only at that point can we start to determine if surgery was a success. | Thank you for your positive feedback on our study. |
| 1) Were there any differences in the patient population, pre-op opioid use, indications for surgery, or technical aspects of the operation that could account for differences in short term recovery trajectory? | Owing to the nature of the study we did not collect additional data to be able to answer your feedback directly, as we focused on data obtained from the patients within the defined eligibility criteria. |
| 2) ) An interesting addition to the study or future study, as the authors have already indicated, would include a final diary entry at long term follow up to reflect on the entire process and see how the long term perception of the postoperative course compares to the short term. | Thank you for this observation. We did continue to collect data to 12 months following surgery, which we are currently analyzing for a further publication as the data identifies different issues when looking long term, as you suggest. |
| 3) It would also be interesting to look at traditional PRO measures such as ODI and see how that correlates with the "construct of recovery". | Yes, this is an interesting observation but not possible from the data we collected and owing to the nature of the study. |
| **Reviewer #2:** | |
| This article attempts to track patient physical, psychological, and social factors and correlate them with patient recovery. The 3 types of recovery meaningful, progressive, and disruptive with typical features are correlated with patient’s diaries of post-operative experiences. This allows for grouping of patient’s under each type. | Thank you for your positive feedback on our study. |
| The study has multiple opportunities to add semi-objective/objective data. By this, I mean mobility monitoring, pain scale/FACES, and functionality scale used to objectively assess patient recovery. This will add more credibility to the paper. The IPA approach is good for diary analysis/grouping however is not sufficient alone. | Yes, this is an interesting observation but not possible from the data we collected.  Also within the qualitative paradigm we would challenge the necessity to include outcome data to add credibility to our findings.  There is much literature within the qualitative paradigm supporting the IPA approach as sufficient without requiring patient outcome data. |
| Further, the overall study lack external validity which is discussed within the paper. The entire population consists of white UK citizens. | Within the qualitative paradigm, external validity is not an important concept. However, the limitations of the generalizability of findings are discussed. |
| This paper is unsalvageable. I recommend the addition of the above comments and more diverse sampling. | We are disappointed to read these comments that appear at conflict with the qualitative paradigm underpinning this paper. |
| **Reviewer #3:** | |
| This study is very well done however I would like to see one change, I would like a chart representing the parameters you outlined which were in their journals (pain, recovery, family support etc) showing how many patients filled each category in order to summarize it all. By doing this it will be clearer to the reader how many patients of the ones with good outcomes fulfilled each category which can lead to further studies. | Thank you and we have created a barchart to illustrate frequencies over time that we hope addresses your point and we agree is a great idea. Please see Fig 2. |

All authors have approved the edited manuscript and submission to PLOS One. We appreciate your time and thank you for considering this manuscript for publication. Should you have any further questions please do not hesitate to contact us.

Kind Regards,

Professor Alison Rushton

On behalf of all authors
